# Ancient Genomes Reveal the Evolutionary History and Origin of Cashmere-Producing Goats in China

**DOI:** 10.1093/molbev/msaa103

**Published:** 2020-04-23

**Authors:** Yudong Cai, Weiwei Fu, Dawei Cai, Rasmus Heller, Zhuqing Zheng, Jia Wen, Hui Li, Xiaolong Wang, Akil Alshawi, Zhouyong Sun, Siqi Zhu, Juan Wang, Miaomiao Yang, Songmei Hu, Yan Li, Zhirui Yang, Mian Gong, Yunan Hou, Tianming Lan, Kui Wu, Yulin Chen, Yu Jiang, Xihong Wang

**Affiliations:** m1 Key Laboratory of Animal Genetics, Breeding and Reproduction of Shaanxi Province, College of Animal Science and Technology, Northwest A&F University, Yangling, China; m2 Research Center for Chinese Frontier Archaeology, Jilin University, Changchun, China; m3 Section for Computational and RNA Biology, Department of Biology, University of Copenhagen, Copenhagen, Denmark; m4 State Key Laboratory for Conservation and Utilization of Subtropical Agro-Bioresources, Guangxi University, Nanning, China; m5 School of Life Sciences, Faculty of Medicine and Health Sciences, University of Nottingham, Nottingham, United Kingdom; m6 Department of Internal and Preventive Medicine, College of Veterinary Medicine, University of Baghdad, Iraqi Ministry of Higher Education and Scientific Research, Iraq; m7 Shaanxi Academy of Archaeology, Xi’an, China; m8 Henan Provincial Institute of Cultural Heritage and Archaeology, Zhengzhou, China; m9 BGI-Shenzhen, Build 11, Beishan Industrial Zone, Yantian District, Shenzhen, China; m10 Laboratory of Genomics and Molecular Biomedicine, Department of Biology, University of Copenhagen, Copenhagen, Denmark; m11 China National GeneBank-Shenzhen, BGI-Shenzhen, China; m12 Cancer Institute, BGI-Research, BGI-Shenzhen, Shenzhen, China

**Keywords:** Chinese goats, ancient DNA, population genomics, adaptation, *FGF5*, *EDA2R*

## Abstract

Goats are one of the most widespread farmed animals across the world; however, their migration route to East Asia and local evolutionary history remain poorly understood. Here, we sequenced 27 ancient Chinese goat genomes dating from the Late Neolithic period to the Iron Age. We found close genetic affinities between ancient and modern Chinese goats, demonstrating their genetic continuity. We found that Chinese goats originated from the eastern regions around the Fertile Crescent, and we estimated that the ancestors of Chinese goats diverged from this population in the Chalcolithic period. Modern Chinese goats were divided into a northern and a southern group, coinciding with the most prominent climatic division in China, and two genes related to hair follicle development, *FGF5* and *EDA2R*, were highly divergent between these populations. We identified a likely causal de novo deletion near *FGF5* in northern Chinese goats that increased to high frequency over time, whereas *EDA2R* harbored standing variation dating to the Neolithic. Our findings add to our understanding of the genetic composition and local evolutionary process of Chinese goats.

## Introduction

As one of the most widespread and adaptable farm animals, goats inhabit a wide agroecological niche spanning all continents. In China, there are 138 million goats, which are distributed among 58 indigenous breeds adapted to various agroclimatic conditions (Du 2011; Skapetas and Bampidis 2016). Due to marked differences in climate, China is often partitioned into a northern and a southern region bounded by the Qinling Mountains-Huaihe River line, which approximates the 0 °C January isotherm and 800 mm isohyet (Jian et al. 2012). Northern China has a relatively cold and dry climate, whereas southern China is relatively hot and humid (Jian et al. 2012). Goats in northern and southern China correspondingly evolved a series of distinct morphological traits (Du 2011). For example, goats in northern China, the main breeding area for cashmere goats, have an extraordinarily dense coat of hair and a more compact body conformation than those in southern China (Liu and Feng 1993; Hai-zhi et al. 2001). These local adaptations of northern and southern goats provide an opportunity to study fine-scale environmental adaptation under the framework of domestication. Understanding the evolutionary genomics behind such adaptations can help animal husbandry meet the challenges of global climate change.

A recent paleogenomic study indicated three distinct Neolithic goat populations around the Fertile Crescent, which contributed differentially to modern goat populations. These include the Neolithic West (Anatolia and the Balkans), Neolithic East (Iran and Turkmenistan), and Neolithic Levant (Jordan and Israel) populations (Daly et al. 2018). However, the origin, genetic turnover, and differentiation of Chinese goats are not well studied at the genomic level. Morphology-based archeological studies show that Chinese domestic goats may have been introduced from the Eurasian steppe to China by the early second millennium BC, concurrent with the gradual cooling and aridification of northern China, which increased the availability of grazing (Liu and Chen 2012a). This time period also included increasing contact between Chinese and Eurasian agricultural civilizations (Yuan et al. 2008; Liu and Chen 2012b). The geographical origin of Chinese goats and the process of their adaptive differentiation are still not clear, however, due to the lack of ancient genomic evidence. Although the origin of Chinese goats has been studied based on mitochondrial sequences (Chen et al. 2005; Han et al. 2010), such studies have severe limitations due to the reduced information content of single-locus analyses.

Time-stamped ancient DNA data can help clarify historical selection processes and provide direct evidence of the genomic dynamics experienced by populations. In this study, we generated genomic data from goat remain dated to ∼3,900–450 years before the present (YBP) from nine archeological sites in China. By comparing the published data on ancient and modern goats, the origin and genetic differentiation of Chinese goats were investigated.

## Results

### Genome Sequencing of Ancient Goats

**Fig. 1.**
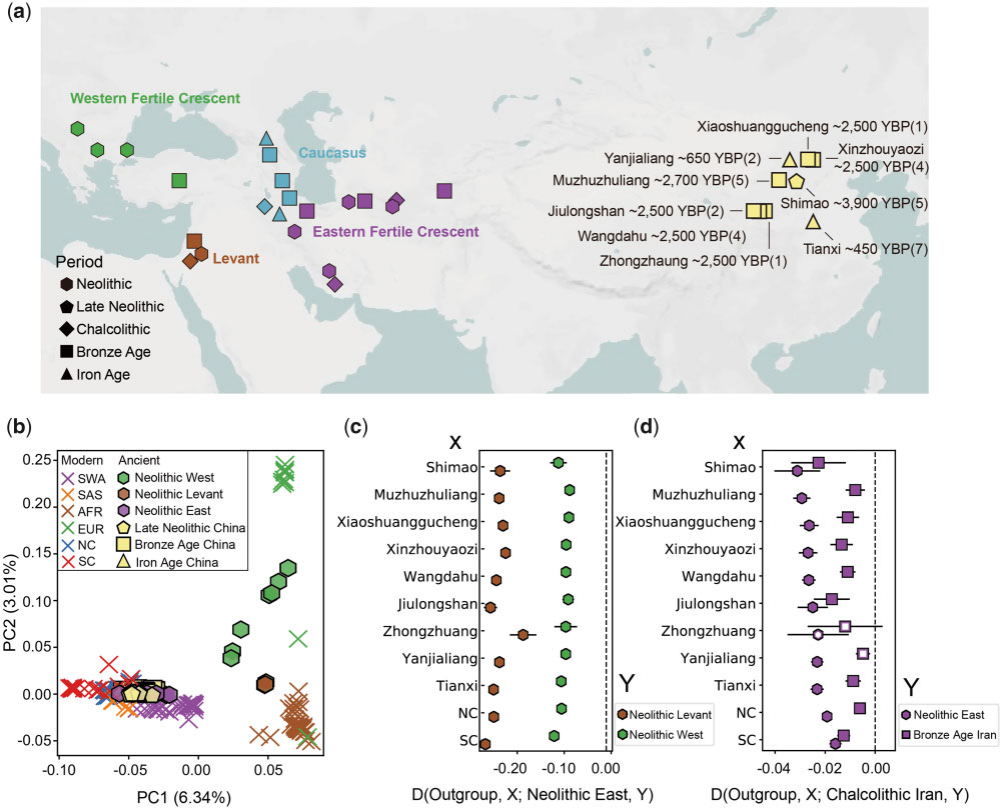
The genetic origin of Chinese goats. (*a*) Locations and ages of all ancient samples used in this study. All ancient Chinese goats are labeled, and the numbers of genomes reported are in parentheses. (*b*) Principal component analysis (PCA) with modern globally distributed goats, ancient Chinese goats, and Neolithic goats around the Fertile Crescent. (*c*) Allele sharing between Chinese goats and Neolithic goats around the Fertile Crescent. A negative *D* statistic indicates a higher level of allele sharing between Chinese goats and Neolithic East goats. (*d*) Allele sharing between Chinese goats and ancient goats from different time periods in the eastern Fertile Crescent. A negative *D* statistic indicates a higher level of allele sharing between Chinese goats and Chalcolithic Iranian goats. Standard errors are shown with bars. Statistics with a |*Z* score| <2 are shown with unfilled symbols.

We generated genome-wide data from 27 sets of Chinese ancient goat remains (table 1), including three sets of goat remains from the Shimao site of the Late Neolithic period (∼3,900 YBP), 16 from six Bronze Age sites (∼2,700–2,500 YBP), and eight from two Iron Age sites (∼650–450 YBP) (fig. 1*a*, supplementary figs. S1, S2 and tables S1, S2, Supplementary Material online). Notably, the Shimao goats represent the oldest known Chinese archaeological samples. We analyzed these data in combination with 54 previously reported ancient goat whole-genome sequencing data sets from the Fertile Crescent (Daly et al. 2018) (supplementary table S3, Supplementary Material online) and China (Zheng et al. 2020) (supplementary table S2, Supplementary Material online). We also added resequencing data from 177 present-day goats around the world (Dong et al. 2015; Alberto et al. 2018; Zheng et al. 2020) (supplementary table S4, Supplementary Material online). All the modern samples were assigned to six geographical groups according to their locations, including Africa (AFR), Europe (EUR), Southwest Asia (SWA), South Asia (SAS), northern China (NC, including 13 breeds), and southern China (SC, including 13 breeds) (supplementary table S5, Supplementary Material online).

**Table 1.** Sample Information of All Ancient Chinese Goats

ID	Site	Date (YBP)	Sex	Mt Hap	Cov. Auto	Cov. Mt
SMG04	Shimao	∼3,900	M	A	0.013	2.788
SMG05	Shimao	∼3,900	F	A	0.007	2.342
SMG07*	Shimao	∼3,900	M	B	0.036	8.208
SMG10	Shimao	∼3,900	M	A	0.097	4.067
SMG11*	Shimao	∼3,900	M	C	0.020	9.730
MZG20	Muzhuzhuliang	**2,740–2,680**	F	A	1.051	41.872
MZG28	Muzhuzhuliang	∼2,700	F	A	0.024	3.344
MZG29	Muzhuzhuliang	∼2,700	F	A	0.181	15.586
MZG34	Muzhuzhuliang	∼2,700	F	A	0.092	5.273
MZG38	Muzhuzhuliang	∼2,700	F	A	0.105	8.602
BG1	Xinzhouyaozi	∼2,500	F	B	0.067	45.689
BG2	Xinzhouyaozi	∼2,500	F	A	0.204	78.515
BG3	Xinzhouyaozi	∼2,500	M	A	0.118	72.911
BG4	Xinzhouyaozi	∼2,500	F	D	0.059	34.689
LSM11	Xiaoshuanggucheng	∼2,500	F	A	0.366	91.485
WDH03	Wangdahu	∼2,500	F	A	0.732	62.431
WDH05	Wangdahu	∼2,500	F	A	0.575	108.729
WDH06*	Wangdahu	∼2,500	F	A	8.106	567.882
WDH08	Wangdahu	∼2,500	M	A	0.038	2.824
JLS05	Jiulongshan	∼2,500	F	C	0.047	2.606
JLS06	Jiulongshan	∼2,500	F	D	0.354	29.336
ZZ01	Zhongzhuang	∼2,500	F	B	0.135	4.226
YJL01	Yanjialiang	∼650	F	A	7.350	661.325
YJL02*	Yanjialiang	**670–625**	M	A	13.439	455.267
GTM01	Tianxi	**438–350**	F	B	0.051	3.439
GTM02	Tianxi	**524–435**	F	B	0.099	13.702
GTM03	Tianxi	**473–308**	F	A	1.524	285.982
GTM04	Tianxi	∼450	F	B	0.020	3.629
GTM06	Tianxi	∼450	M	B	0.202	107.890
GTM08	Tianxi	∼450	F	B	0.181	57.152
GTM11	Tianxi	∼450	F	B	0.189	57.842

Note.—Samples marked with an asterisk were previously published (Zheng et al. 2020). “Mt Hap,” mitochondrial haplogroup; “Cov. Auto,” mean depth of coverage across autosomes; “Cov. Mt,” mean depth of coverage across mitochondria. Calibrated radiocarbon dates are shown in bold with 95.4% confidence interval. Dates in plain text are estimated from the archaeological context or the radiocarbon dates from the same site (Supplementary Material online). Note that the majority of Muzhuzhuliang belong to the Longshan culture, similar to the Shimao site, while a few remains are more recent. Five Muzhuzhuliang samples were collected in this study. One of them, MZG20, was radiocarbon dated to ~2700 YBP. Therefore, the other four samples in this site without radiocarbon-dated are also marked as ~2700 YBP (Supplementary Material online).

### The Origin of Chinese Goats

To explore the global genetic structure of goats, we performed principal component analysis (PCA) on all the modern populations and projected ancient goats onto those components. The modern samples were divided into geographic subgroups, with the Asian, European, and African samples representing three distinct apices (fig. 1*b*, supplementary fig. S3, Supplementary Material online). All the ancient Chinese goats clustered with modern Chinese goats, demonstrating their genetic continuity from the Late Neolithic to the present. To explore the Neolithic origin of Chinese goats, we also investigated the relationships between Chinese goats and the three Neolithic populations around the domestication center. Our results showed that both modern and ancient Chinese goats cluster with the Neolithic population from the eastern part of the Fertile Crescent (Neolithic East) (fig. 1*b*). These relationships were also confirmed by the phylogenetic tree (supplementary fig. S4, Supplementary Material online).

To further determine the origin of Chinese goats, we then used *D* statistics to measure the genetic affinities among all Chinese goats and the Neolithic goats around the Fertile Crescent (fig. 1*c*). As with the above analyses (fig. 1*b* and supplementary fig. S4, Supplementary Material online), among the three Neolithic populations around the Fertile Crescent, both ancient and modern Chinese goats show the highest level of allele sharing with Neolithic East goats (fig. 1*c*). Furthermore, among eastern Fertile Crescent goats from different periods, samples from ∼7,000 to 6,000 YBP (Chalcolithic period) show more genetic affinity with Chinese goats (fig. 1*d* and supplementary fig. S5, Supplementary Material online), and Chinese goats have a closer genetic affinity with these ancient goats than with other contemporary goats (supplementary fig. S6, Supplementary Material online).

### The Genetic Differentiation of Chinese Goats

**Fig. 2.**
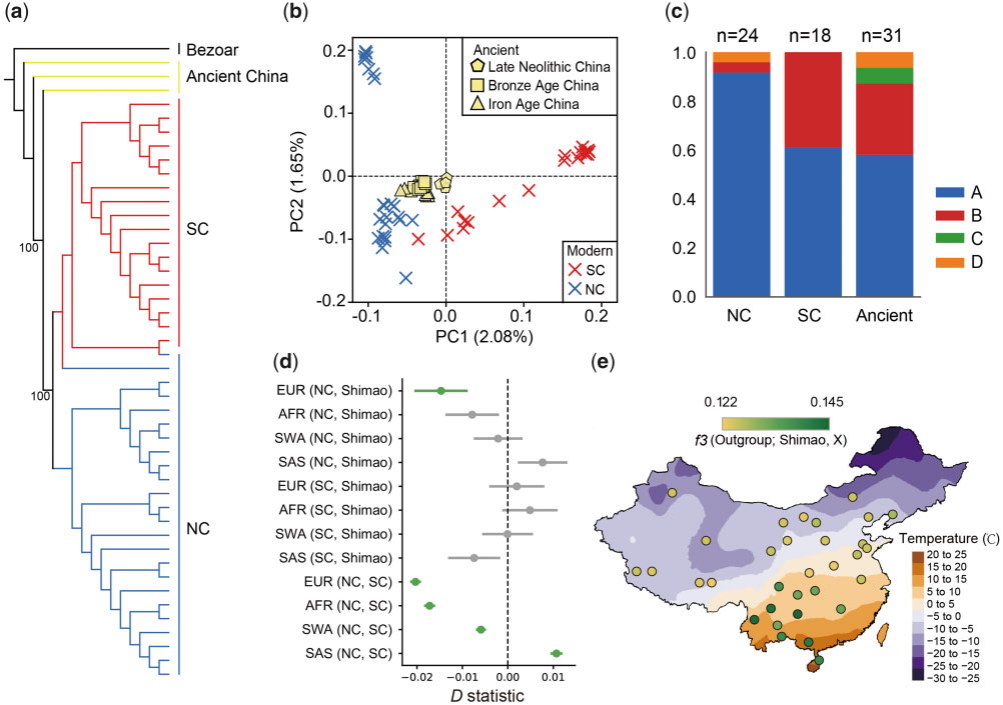
Genetic differentiation in Chinese goats. (*a*) Neighbor-Joining (NJ) tree of the Chinese goat population, only modern samples and three ancient (YJL01, YJL02, and WDH06) Chinese goats with at least 3× coverage were included. (*b*) Principal component analysis (PCA) including all Chinese goats. The ancient samples were projected onto the axes computed using modern populations. All modern samples are represented with crosses, and ancient samples are shown with other symbols according to their age. (*c*) Mitochondrial haplogroup distributions in different Chinese goat groups. (*d*) In each test X (Y, Z), a positive *D* statistic indicates a higher level of allele sharing between X and Z, whereas a negative one indicates a higher level of allele sharing between X and Y. Points with a |*Z* score| >2 are colored in green. (*e*) Outgroup *f*3 statistics for the shared genetic history between ancient Shimao goats and modern Asian goats. Higher *f*3 values represent a closer genetic affinity with Shimao. The average temperature in January is also shown.

To focus on the diversity within China, we then performed phylogenetic analysis and PCA using only Chinese goats. The phylogenetic analyses using modern Chinese goats and three ancient samples (YJL01 and YJL02 from Yanjialiang, WDH06 from Wangdahu) with at least 7× coverage (7.350×, 13.439×, and 8.106×, respectively) show that ancient Chinese goats are basal to the lineage of all Chinese goats, whereas modern Chinese goats form a monophyletic group with a north–south structure (fig. 2*a*). This topology was supported by PCA with additional ancient samples, including low-depth samples. All the ancient Chinese goats were located in the center of the modern Chinese goats, and the first component was driven by the difference between NC and SC (fig. 2*b*), which also coincides with the results of the fineSTRUCTURE and TreeMix analysis (supplementary figs. S7 and S8, Supplementary Material online). We then calculated the nucleotide diversity (*θ*_π_) within each group and the inbreeding coefficient (*F*) for each individual (supplementary figs. S9 and S10, Supplementary Material online). The results showed that modern SC has lower diversity (Student’s *t*-test *P *=* *2.14 × 10^−24^) and a higher inbreeding coefficient (Student’s *t*-test *P *=* *0.0035) than NC. The divergence time between NC and SC as estimated by diffusion approximations for demographic inference (∂a∂i) was ∼3,013 YBP (1,947–6,059 YBP, 95% CI) (supplementary fig. S11, Supplementary Material online). These results confirmed that modern Chinese goats were mainly descended from ancient Chinese goats with a subsequent north–south separation.

The genetic differentiation within Chinese goats is also reflected in mitochondrial DNA. The maternal phylogenetic analysis showed that ancient Chinese goats were highly diverse, with four haplogroups (58% A, 29% B, 6% C, and 6% D) (fig. 2*c*). This diverse mtDNA gene pool has existed since the Late Neolithic, with three haplogroups (A–C) found in Shimao (supplementary fig. S12, Supplementary Material online). In modern Chinese goats, SC has a similar haplotype A frequency (61%) to that of ancient Chinese goats, which is significantly lower than that of NC (92%, Fisher’s exact test *P *=* *0.02) and the previously reported worldwide haplogroup A frequency (Naderi et al. 2007) (fig. 2*c*). The remaining SC goats fall within the B haplogroup, with a significantly higher frequency than that of NC (Fisher’s exact test *P *=* *0.01) (fig. 2*c*).

We then calculated outgroup *f*3 statistics to measure the genetic affinities between the global population of ancient goats and modern Chinese goats. The results showed that among all ancient populations, modern Chinese goats have higher genetic affinity to ancient Chinese goats (supplementary fig. S13, Supplementary Material online), which further corroborates the genetic continuity of Chinese goats rather than indicating population turnover due to immigration. However, compared with SC, NC exhibited less allele sharing with ancient Chinese goats from all periods (fig. 2*e* and supplementary fig. S14, Supplementary Material online), and this result was also supported by *D* statistics (supplementary fig. S15, Supplementary Material online). To detect possible gene flow between modern Chinese and non-Chinese populations, we calculated *D* statistics (Outgroup, X; NC/SC, Shimao) using modern global goats as the query population (X) (fig. 2*d*). The results show that EUR shares more alleles with NC than with Shimao and SC (fig. 2*d*). Furthermore, we used an approximate Bayesian computation (ABC) approach to compare four demographic scenarios with or without gene flow from EUR to NC (supplementary fig. S16, Supplementary Material online). The results show that the admixed model has a high posterior probability (0.89), in line with the *D* statistics, suggesting that NC may be affected by gene flow from EUR.

### Local Adaptation by Selective Sweeps

**Fig. 3.**
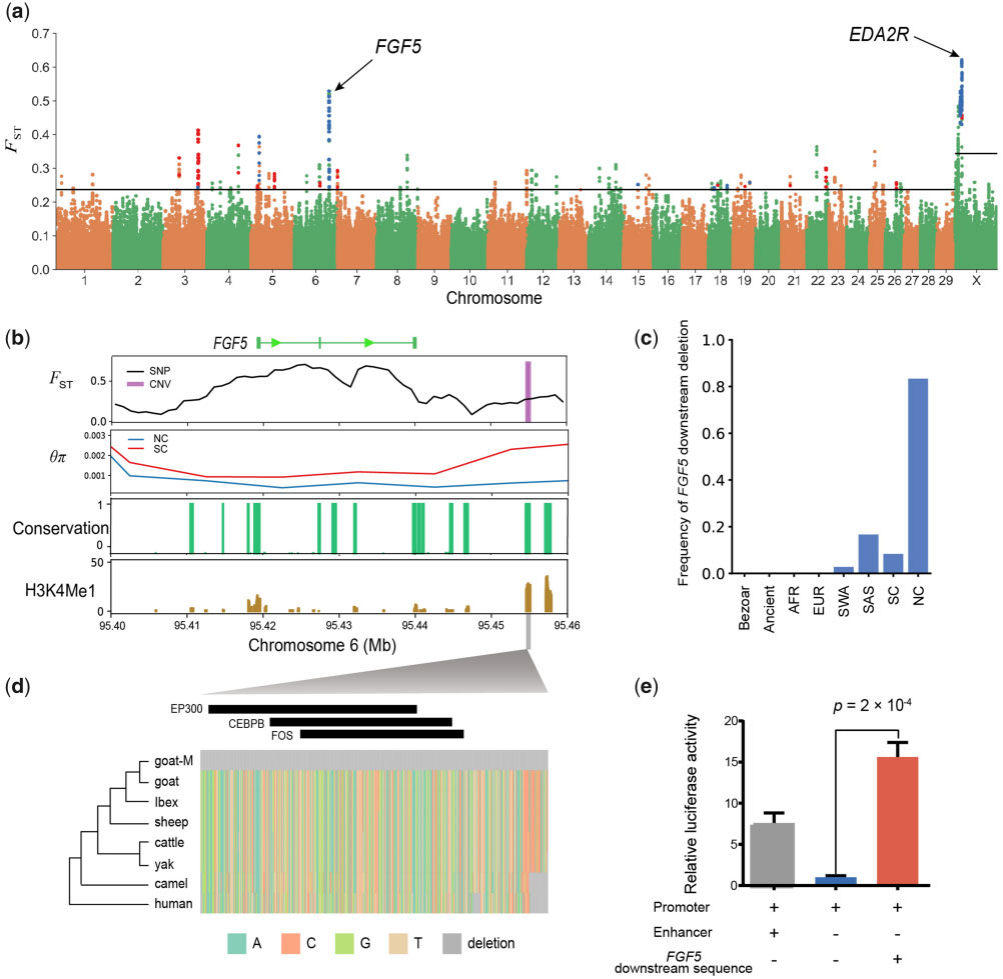
Genome-wide selection scan. (*a*) Manhattan plot of the genome-wide distribution of pairwise *F*_ST_ between SC and NC using a 50-kb window size and a 10-kb step size. The threshold of *F*_ST_ values is marked with a horizontal line. Windows selected in NC and SC are colored in blue and red, respectively. (*b*) Selection signs around *FGF5*. *F*_ST_ based on single nucleotide polymorphisms (SNPs) is plotted as a line using a nonoverlapping 10-kb sliding window. *F*_ST_ based on copy number variants (CNVs) is plotted as a rectangle. The conservation scores of 100 vertebrate species are shown in green, and H3K4Me1 signals are shown in brown. (*c*) The frequency of the 504-bp deletion (chromosome 6: 95,454,685–95,455,188 bp) in each population. (*d*) Sequence context of the 504-bp deletion in different species, showing deletion solely in the goat-mutant (goat-M) type. The black rectangles indicate transcription factor binding sites. (*e*) Dual-luciferase assay using goat fibroblasts showing that the *FGF5* downstream deletion sequence enhanced the activity of luciferase. Data are shown as the mean ± standard error. The *P*-value was calculated using Student’s *t*-test.

Northern and southern Chinese goats inhabit divergent environments and have separated with respect to both genotype and phenotype (Du 2011). For example, 11 of the 19 local goat breeds in northern China are cashmere-producing, whereas none of the 39 southern breeds are cashmere-producing (Du 2011). To gain insight into the genetic basis of the north–south divergence in modern Chinese goats, we next scanned for selection signatures separately in NC and SC. We calculated *F*_ST_ and the *θ*_π_ ratio between NC and SC and used an outlier approach to identify genomic regions undergoing selective sweeps in these two groups. A total of 24 selective sweep regions spanning 33 candidate genes were identified (supplementary fig. S17 and table S6, Supplementary Material online). The top two *F*_ST_ outliers contained two genes, Fibroblast Growth Factor 5 (*FGF5*) on chromosome 6 and Ectodysplasin A2 Receptor (*EDA2R*) on chromosome X (fig. 3*a*), both of which are related to the development of hair follicles (Botchkarev and Fessing 2005; Zhang et al. 2009; Wang, Cai, et al. 2016). Furthermore, several other genes that show high selection signals are plausibly related to environmental adaptation. For example, *MAGED1* (selected in NC) can bind to nuclear receptor RORα and thereby affect circadian clock function (Wang et al. 2010). *CDC25A* (selected in SC) is related to body size in goats (Wang, Liu, et al. 2016). *SPAG17* (selected in NC) is associated with human adult height (Weedon et al. 2008), and a known mutation can produce mice with significantly shorter hindlimb length (Teves et al. 2015), consistent with selection for contracted limbs in cold-adapted populations (Allen 1877).

The strongest selection signal found on the autosomes (chromosome 6: 95,400–95,640 kb) harbors the *FGF5* gene (fig. 3*a*), which is related to the regulation of hair length in mice, dogs, and humans (Mizuno et al. 2011; Dierks et al. 2013; Higgins et al. 2014). In goats, a previous CRISPR/Cas9-mediated animal experiment showed that the disruption of *FGF5* resulted in more secondary hair follicles and longer fibers, resulting in increased cashmere production (Wang, Cai, et al. 2016). We validated the expression of *FGF5* in the outer root sheaths of follicles in cashmere goats by immunohistochemical experiments (supplementary fig. S18, Supplementary Material online). Therefore, *FGF5* appears to negatively regulate the development of hair follicles.

To identify the potential causal mutation around the *FGF5* locus, we inspected all of the divergent mutations between NC and SC. There were no missense mutations in coding regions, but a 504-bp (chromosome 6: 95,454,685–95,455,188 bp) deletion was detected ∼14 kb downstream of *FGF5* (fig. 3*b*). The frequency of this deletion was high in NC (83.3%) but much lower in other Asian goats (SC: 8.3%, SAS: 16.7%, SWA: 2.8%). This deletion is absent in European and African goats, as well as in all bezoars (*Capra aegagrus*) (fig. 3*c*). Furthermore, all 19 Chinese cashmere goats in this study carried this mutation, with 14 homozygous animals and 5 heterozygous animals. Notably, none of the ancient goats collected in this study (in China and the Near East regions) were found to harbor this deletion (supplementary note 15, Supplementary Material online).

Multiple lines of evidence suggest that this deletion in NC may have an enhancer function with regards to *FGF5*. It is located in a highly conserved element across 100 vertebrate species (Casper et al. 2018) and overlaps a H3K4Me1 peak (usually enriched at enhancers [Rada-Iglesias 2018]) in NHEK cells according to ENCODE (Encyclopedia of DNA Elements) data (Rosenbloom et al. 2012) (fig. 3*b*). NHEK cells are derived from normal human epidermal keratinocytes, which are used to replace hair follicle cells. Furthermore, this deletion region harbors three transcription factor binding sites (EP300, FOS, and CEBPB) verified in the human genome (Wang et al. 2013), of which FOS is associated with apoptotic cell death, possibly controlling the hair follicle cycle (Fisher et al. 1991) (fig. 3*d*). To confirm this, we cloned this 504-bp deletion sequence into a luciferase reporter vector (pGL3-Promoter) and transfected it into goat (fig. 3*e*) and sheep (supplementary fig. S19, Supplementary Material online) fibroblasts. The results show that the *FGF5* downstream sequence led to a significant increase in luciferase expression (Student’s *t*-test *P *=* *2 × 10^−4^ and 2 × 10^−5^, respectively) compared with the promoter-only construct (fig. 3*e* and supplementary fig. S19, Supplementary Material online).

**Fig. 4.**
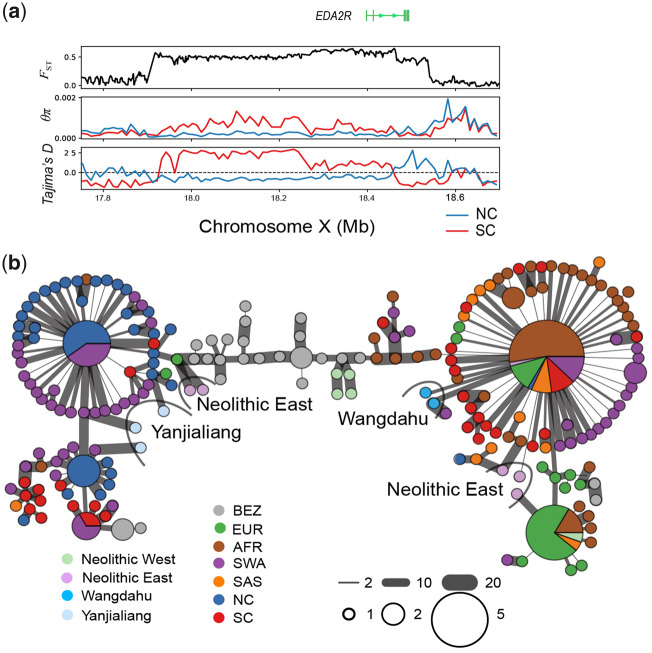
Selective sweep region on the X chromosome. (*a*) Selection signals around *EDA2R* in X chromosome (NW_017189516.1). *F*_ST_, *θ*_π_, and Tajima’s *D* were plotted as a line using a nonoverlapping 10-kb sliding window. (*b*) Haplotype network based on pairwise differences within the selective sweep region (chrX: 17,915,001–18,539,000) in *EDA2R*.

Another region with extremely high *F*_ST_ occurs on the X chromosome at 17,915–18,539 kb (fig. 3*a*). Low *θ*_π_ and Tajima’s *D* values confirmed this selective sweep in NC (fig. 4*a*). This 624 kb region contained only one gene, *EDA2R* (also known as *XEDAR*), which has been reported to regulate primary hair follicle placode formation (Botchkarev and Fessing 2005; Zhang et al. 2009) and is associated with male pattern hair loss (Prodi et al. 2008). The expression of *EDA2R* in northern Chinese cashmere goats exhibits a seasonal pattern, being mainly expressed from August to October, and has a high correlation with *FGF5* expression (*R*^2^ = 0.8, *P *=* *0.007). It may, therefore, be related to seasonal cold adaptation (supplementary fig. S20, Supplementary Material online). The haplotype network of the *EDA2R*-selected region showed two highly divergent haplogroups in worldwide domestic goats (fig. 4*b*). Of the NC individuals, 95.3% belong to one haplogroup (hereafter referred to as the NC-type). This haplotype is also found in SC and SWA, but at lower frequencies (36% and 57%, respectively). We traced the emergence of the NC-type among all available ancient and modern samples. At the early domestication stage, the NC-type was already present in the eastern Fertile Crescent (fig. 4*b*). Furthermore, the NC-type also existed in Uzbekistan and Turkmenistan in the post-Neolithic period. Using all the ancient Chinese samples, we observed that the frequency of the NC-type in the Iron Age (78.6%) was significantly higher than that in the Bronze Age (33.3%) (Fisher’s exact test *P *=* *0.009) (supplementary fig. S21, Supplementary Material online). These results suggest that the NC-type of *EDA2R* descended from an ancestral standing variant >8,000 years old and then spread into Asian goat populations (NC, SC, and SWA).

## Discussion

Time-stamped ancient goat DNA data from China provide novel opportunities to investigate the evolutionary origin and genetic differentiation of Chinese goats. Our paleogenomic analyses, including phylogenetic analysis using high-coverage samples (fig. 2*a*) in addition to PCA and outgroup *f*3 using all the ancient Chinese samples (figs. 1*b* and 2*b* and supplementary fig. S13, Supplementary Material online), demonstrate the genetic continuity of Chinese goats from the Late Neolithic to the present. In addition, among three distinct Neolithic goat populations located around the domestication center, Chinese goats have a closer genetic affinity with goats in the eastern Fertile Crescent than with those in other regions (fig. 1*b* and *c*), demonstrating that the Neolithic origin of Chinese goats was the eastern Fertile Crescent. Mitochondrial haplogroup A was mainly distributed in the western regions around the Fertile Crescent in the Neolithic period and then dispersed to other regions after the Neolithic (Daly et al. 2018). The presence of haplogroup A at the Shimao site (supplementary fig. S12, Supplementary Material online) suggests that the ancestor of Chinese goats likely left the eastern Fertile Crescent after the Neolithic period. Furthermore, among the ancient goat populations of different periods in the eastern Fertile Crescent, we observed that goats from the Chalcolithic period show more genetic affinity with Chinese goats than those from the Neolithic (sample dates ranging ∼9,000–8,000 YBP) or Bronze Age (sample dates ranging ∼4,400–3,900 YBP) (fig. 1*d*, supplementary figs. S5 and S6, Supplementary Material online). On the basis of the above, we propose that Chinese goats are derived from a stock that separated from the eastern Fertile Crescent population between ∼8,000 and 4,400 YBP, roughly corresponding to the Chalcolithic period.

Modern Chinese goats showed north–south genetic differentiation in both the nuclear (fig. 2*a* and *b*) and mitochondrial (fig. 2*c*) genomes, and southern Chinese goats maintained a gene pool more similar to that of ancient Chinese goats (fig. 2*c*–*e*). The gene flow from EUR into NC (fig. 2*d* and supplementary fig. S16, Supplementary Material online) may contribute to this divergence. The higher level of allele sharing between goats in northern China and Europe, in line with the north-to-south decline of European gene flow observed in East Asian humans (Qin et al. 2015), suggested a northern path of gene flow from Europe to East Asia. The diffusion of goats with European ancestry into northern China may be associated with the recent introgression of Mongolian people into extensive areas of China (May 2012); this migration could also have been the driving force behind the exotic introgression of Eurasian taurine cattle into northern China (Chen et al. 2018).

By performing whole-genome selection scans, we analyzed the genetic basis of the divergence between modern Chinese goat populations. The top two divergent genes between NC and SC, a primary hair follicle-related gene (*EDA2R*) and a secondary hair follicle-related gene (*FGF5*) are both involved in hair growth. *FGF5* is an inhibitor of the hair anagen phase and knocking out this gene can increase cashmere production (Wang, Cai, et al. 2016). We reported a downstream deletion of *FGF5* that carries potential cis-regulatory enhancer regions (fig. 3*b*, *d*, and *e*). This deletion was found in all Chinese cashmere goats and had a high frequency in NC goats compared with goats in other areas (fig. 3*c*). These results indicate that this deletion may promote cashmere production by reducing *FGF5* expression.

Ancient DNA provides direct insight with which to clarify historical selection processes. We searched for the source of the *FGF5* downstream deletion but did not find it in any of the ancient samples (supplementary note 15, Supplementary Material online). Although our sample size does not allow us to exclude that the deletion did, in fact, exist in historical times, our result is compatible with the deletion emerging as a de novo mutation within the last 450 years. Therefore, *FGF5* may represent a case of rapid gain of function by cis-regulatory element evolution, a process that has previously been established in Yakutian horses (Librado et al. 2015). The evolutionary trajectory of *EDA2R* is different. The NC-type of *EDA2R* in Chinese goats appears to have descended from an ancestral standing variant of ancient goats in the eastern Fertile Crescent (fig. 4*b*) and then undergone a significant increase in frequency in northern China during the Iron Age (sample dates ranging ∼650–450 YBP) (supplementary fig. S21, Supplementary Material online).

As wool is an important animal product with a long history of utilization by humans in China (Zhao and Jin 2007), the evolution of both *FGF5* and *EDA2R* is likely to have been driven by artificial selection. Furthermore, the selected haplotypes of these two genes emerged or increased roughly contemporaneously with the period of large-scale cooling in the Northern Hemisphere (supplementary fig. S22, Supplementary Material online), known as the Little Ice Age (∼600–300 YBP) (Mann et al. 2009). Hence, it is possible that decreasing temperatures motivated an increase in artificial selection for wool production in the cooler northern parts of China. This may represent a case of environmental change strengthening the human-induced selection regime for wool production traits. Such dynamic selection pressures during domestication and husbandry is an interesting and understudied topic worthy of further investigation.

In conclusion, through an analysis of worldwide ancient and modern goat genomes, we demonstrated that Chinese goats are mainly descended from Chalcolithic eastern Fertile Crescent goats with little genetic turnover from their first arrival in China to the present day. Modern goats in southern China in particular have retained a relatively archaic genetic profile, whereas northern Chinese goats show shifts in both nuclear and mitochondrial DNA over time. The two most divergent genes between NC and SC are both related to hair follicle development, and the evolutionary trajectories of these genes were uncovered through ancient DNA. Our study reveals the genetic origin and genetic differentiation of Chinese goats and contributes to a new understanding of the eastward dispersal of domesticated goats.

## Materials and Methods

### Ancient Samples Sequencing

DNA was extracted from 27 ancient bone and teeth samples in a dedicated ancient DNA laboratory at Jilin University using a modified silica-spin column method. A genomic DNA library was prepared from 55.5 µl of ancient DNA using NEBNext Ultra DNA Library Prep Kit for Illumina (New England Biolabs Inc.). Then, all bar-coded libraries were sequenced on an Illumina HiSeq X Ten platform (paired-end 150 bp). Besides, we downloaded published genomic data of worldwide modern and ancient goats from the NCBI deposit (supplementary tables S3 and S4, Supplementary Material online).

### Processing and Alignment of Sequencing Reads

After a series of quality controls (supplementary note 3, Supplementary Material online), the cleaned reads were aligned against the most recent goat reference genome (ARS1, GCF_001704415.1) (Bickhart et al. 2017) using BWA-backtrack (BWA aln) algorithm (Li and Durbin 2010). The DNA damage pattern was characterized using mapDamage (Jónsson et al. 2013). The SNP calling was performed using GATK (McKenna et al. 2010), and the genotype likelihoods were estimated using ANGSD (Korneliussen et al. 2014). The transition sites were excluded in ancient samples to minimize the false-positive results.

### Population Genetics Analysis

For phylogenetic analysis, we use modern and ancient goats with at least 3× sequence depth. MEGA X (Kumar et al. 2018) was employed to construct the Neighbor-Join tree with 100 bootstraps (supplementary note 7, Supplementary Material online). Then, iTOL (Letunic and Bork 2016) was used to visualize the topological structure. We employed smartpca implemented in the EIGENSOFT package to perform PCA analysis (Patterson et al. 2006), using the “lsqproject” and “autoshrink” options (supplementary note 6, Supplementary Material online). The outgroup f3 statistics were calculated using AdmixTools (Patterson et al. 2012) and with Qazvin Bezoar (*C. aegagrus*) used as outgroup (supplementary note 9, Supplementary Material online). The *D* statistics were calculated via ANGSD (Soraggi et al. 2018) with argali (*Ovis ammon*) as the outgroup (supplementary note 9, Supplementary Material online).

### Demographic History Analyses

We inferred the demographic history for Chinese goats using ∂a∂i (Gutenkunst et al. 2009). The site frequency spectra used in ∂a∂i was computed from the total of 454 Mb sequence of the 11 NC and 11 SC (supplementary note 12, Supplementary Material online). A simple model was fit first, then the complexity of the model was increased gradually (supplementary fig. S11, Supplementary Material online). The best model was selected according to the likelihoods and Akaike’s information criterion, and nonparametric bootstrapping (100 times) was performed to determine the confidence interval of each parameter. Furthermore, we compared four hypothesized models (supplementary fig. S16, Supplementary Material online) with three groups (EUR, NC and SC) using ABC approach implemented in DIYABC (Cornuet et al. 2014). We performed four million simulations for each scenario using 6,497 SNPs which thinned using a distance filter of interval >50-kb and a rare SNP filter of MAF >0.05. All one-sample and two-sample summary statistics were used.

### Genome-Wide Selection Analysis

To detect regions under selection, several statistics including *F*_ST_, *θ*_π_, and Tajima’s *D* were calculated with a 50-kb sliding window and 10-kb step size via vcftools (Danecek et al. 2011). The outlier windows with high *F*_ST_ value were first retained (supplementary note 10, Supplementary Material online). Then, according to the *θ*_π_ value in each group, we classified the windows as being under selection in either NC or SC (supplementary fig. S17, Supplementary Material online). The X chromosome in the goat reference genome (ARS1) was separated into two scaffolds: NW_017189516.1 and NW_017189517.1. To avoid ambiguity, we concatenated these two scaffolds into a continuous one (NW_017189516.1 is before NW_017189517.1) in the Manhattan plot (fig. 3*a*), referred to as X.

### Immunohistochemistry Staining

According to the manufacturer’s instructions, Paraffin-embedded tissue was deparaffinized, and then antigen retrieval was carried out by treatment with EDTA antigen repair buffer (pH = 9.0). Tissues were immersed in 3% hydrogen peroxide for 25 min to eliminate the endogenous peroxidase activity and blocked with blocking reagent in 3% BSA for a further 30 min. Then, samples were incubated at 4 °C overnight in a humidified chamber with the following primary antibody: *FGF5* (rabbit, 1:300; Abcam, UK). After being washed three times with PBS for 5 min each time, these samples were treated with a secondary antibody (HRP marker) for 50 min at room temperature. Later, samples were rinsed several times with PBS, treated with DBA, counterstained with hematoxylin, and imaged with light microscopy (supplementary fig. S18, Supplementary Material online).

### Dual-Luciferase Reporter Analysis

The CNV sequence downstream *FGF5* gene of goats was amplified through PCR. Then the sequence was inserted into the pGL3 luciferase reporter vector (Promega) and confirmed by sanger sequencing. The transfection was performed using TurboFect (R0531, Thermo Scientific, Waltham, USA). After 24 h of transfection, the activity of luciferase was measured with the Dual-Luciferase Reporter Assay System (Promega). Both goat and sheep fibroblast cells were used in this assay. Each experiment was independently performed at least three times (fig. 3*e* and supplementary fig. S19, Supplementary Material online).

## Supplementary Material

Supplementary data are available at *Molecular Biology and Evolution* online.

## Supplementary Material

msaa103_Supplementary_Data
