# Supplementary material for: Ancient Genomes Reveal the Evolutionary History and Origin of Cashmere-Producing Goats in China
Source: Mol Biol Evol. 2020 Apr 23;37(7):2099–109. doi: 10.1093/molbev/msaa103 (PMC7306693; doi:10.1093/molbev/msaa103)
Supplement: msaa103_Supplementary_Data [file msaa103_supplementary_data.pdf]

Supplementary Information for

Ancient genomes reveal the evolutionary history and origin of Cashmere producing goats in China

Yudong Cai[1†], Weiwei Fu[1†], Dawei Cai[2†], Rasmus Heller[3], Zhuqing Zheng[1], Jia Wen[1], Hui Li[1,4], Xiaolong Wang[1], Akil Alshawi[5,6], Zhouyong Sun[7], Siqi Zhu[2], Juan Wang[8], Miaomiao Yang[7], Songmei Hu[7], Yan Li[1], Zhirui Yang[1], Mian Gong[1], Yunan Hou[1], Tianming Lan[9,10], Kui Wu[11,12], Yulin Chen[1], Yu Jiang[1*], Xihong Wang[1*]

[†]These authors contributed equally to this work.

*Corresponding author

Email: yu.jiang@nwafu.edu.cn (Yu Jiang); wxh@nwafu.edu.cn (Xihong Wang)

This PDF file includes:

Supplementary text
Figures S1 to S24
Tables S1 to S8
References

Supplementary Note 1: Archaeological background

1.1 Shimao, Shaanxi, China

Shimao is located in Shimao Village, Gaojiabao Town, Shenmu County, Yulin City, Shaanxi Province. A combined archaeologists team, which was composed of the Shaanxi Provincial Institute of Archaeology, the Yulin Cultural Relics, Archaeological Exploration Team, and the Shenmu Bureau of culture, conducted a rescue archaeological excavation of the ruins during 2012 to 2013 (Sun et al. 2015). Through the identification of unearthed jade, pottery, and other artefacts, it is shown that the Shimao is a super large central settlement (larger than 100 ha) dating to time from late Longshan Culture to early Xia Dynasty (from 4300 to 3800 years ago), which played a central role in the spiritual and political world among agro-pastoralists of the north Loess Plateau region (Sun et al. 2018) and is of great significance for studying the origin of Chinese civilization. Thousands of faunal remains were unearthed from the Shimao, suggesting an emerging agro-pastoralist tradition in the north frontier of China (Hu et al. 2016; Owlett et al. 2018). Among them, domestic animal bones mainly include sheep, goats, cattle, pigs, dogs, and horses. From the use of animal resources in Shimao, humans were engaged in animal husbandry, agriculture, and hunting at that time. So the economic type during that period should be in the form of semi-agricultural and semi-pastoral, with a small amount of hunting (Hu et al. 2016). Several ancient cattle samples have been radiocarbon dated to about 3900 YBP (N. Chen et al. 2018). Four samples from Shimao were used in this study, including three newly sequenced samples: SMG04, SMG05, and SMG10, as well as two published samples: SMG07 and SMG11.

1.2 Xiaoshuanggucheng, Inner Mongolia, China

Xiaoshuanggucheng cemetery is located at about 1 km southeast of Xiaoshuanggucheng Village, which belongs to Liangcheng County and was excavated by the Inner Mongolian Institute of Cultural Relics and Archaeology from May to October in 2003. It is at the north of the Daihai Lake and on the northern slope of the Wangmu Mountain. In August 2003, in order to cooperate with the construction of Daihai Power Plant, the Inner Mongolia Cultural Relics and Archaeology Institute team conducted a rescue archaeological excavation of this cemetery. A total of 14 tombs were discovered. Among them, 11 tombs have animal remains. The number of sheep and goats is the highest, followed by cattle, and finally horses, but dogs and pigs are not seen. Xiaoshuanggucheng had the characteristics of the nomadic economy. According to the features of the excavated artifacts and remains, the age of this cemetery is approximately 2500 years old (Chen and Cao 2006), roughly from the late Spring and Autumn Period to the early Warring States Period. One sample (LSM11) from Xiaoshuanggucheng was used in this study.

1.3 Xinzhouyaozi, Inner Mongolia, China

Xinzhouyaozi cemetery is located at about 2 km northeast of Bancheng Village, which also belongs to Liangcheng County and was excavated by the Inner Mongolian Institute of Cultural Relics and Archaeology from May to October in 2003. It is at the west of the

Daihai Lake and 3 km east of the Maoqinggou cemetery. The cemetery covers an area of about 15,000 square meters. There are gullies in the southeast and northwest parts of the cemetery, and some of the tombs were destroyed by the gullies. The Inner Mongolia Institute of Cultural Relics and Archaeology conducted a rescue archaeological excavation of the cemetery. A total of 67 tombs were discovered. There are 43 tombs in the cemetery with animal remains, including horses, cattle, goats, sheep, dogs, and pigs. According to the statistical results of the site, the number of goats is the highest, followed by sheep and cattle, while the number of horses, pigs, and dogs is the least. According to the characteristics of excavated artifacts and remains, the age of the site is approximately 2500 years old (Chen and Cao 2006), roughly from the late Spring and Autumn Period to the early Warring States Period. Four samples from Xinzhouyaozi cemetery were used in this study, including BG1, BG2, BG3, BG4.

1.4 Yanjialiang, Inner Mongolia, China

Yanjialiang is an archaeological site of Yuan Dynasty co-excavated by Inner Mongolia Institute of Cultural Relics and Archaeology and Baotou Heritage Management Office in 2006 (Ta et al. 2010). This site, with a total area of 2000 square meters, is located on the south side of Yanjialiang Village, Machi County, Jiuyuan borough, Baotou City. The Yanjialiang is an important archaeological excavation of Yuan Dynasty and provides significantly important reference for the study of the inhabitants' livelihood and production of the Yuan Dynasty. There are more than ten thousands of objects made of different materials are excavated, especially blue-and-white porcelain, and a large number of animal bones. Two samples from Yanjialiang were used in this study, including a newly sequenced sample (YJL01) and a published sample (YJL02). The YJL02 was radiocarbon dated to about 650BP.

1.5 Muzhuzhuliang, Shaanxi, China

Muzhuzhuliang is located at about 3 km south of Yejihe Village, Dabaodang Town, Shenmu County, Shaanxi Province. From 2011 to 2012, due to the construction of the DabaoDang yushen Industrial Resettlement, the Shaanxi Provincial Institute of Archaeology, the Yulin Cultural Relics and Archaeological Exploration Team jointly conducted archaeological exploration and excavation of the ruins. The Muzhuzhuliang is one of the branches of late Longshan Culture (Wang et al. 2015). The Muzhuzhuliang provides basic information on the arrangement, scale, and production of this kind of settlement. The excavated human remains, faunal remains and palaeobotanic remains provide materials to investigate the subsistence, palaeo-environment, and lifeways during the occupation of the site. Five samples from Muzhuzhuliang were used in this study, including MZG20, MZG28, MZG29, MZG34, and MZG38. Among them, MZG20 was radiocarbon dated to about 2700 YBP. According to the radiocarbon date of MZG20, we regard all samples in this site to ~2700 YBP and thus classified the period of ancient goats in this site to Bronze Age in this study.

1.6 Wangdahu, Ningxia, China

WangDahu Cemetery is located in the northeastern part of WangDahu Village, Gucheng Town, Pengyang County. It is about 15 kilometers south of the Gucheng town and 35 kilometers away from Pengyang County. During July 2007, rescued excavations were carried out due to the theft of the cemetery. A total of seven tombs in the Spring and Autumn Period and the Warring States Period were discovered. Each of the tombs was buried with horses, cattle, and sheep skulls. The date of Wangdahu site is approximately at ~2,500 years ago (Zhao et al. 2010). Many bronze daggers and dagger-ax were found but didn't include household tools. It is speculated that the owner here belongs to the nomads. Four samples from Wangdahu were used in this study, including three new sequenced examples: WDH03, WDH05, WDH08, and one published sample WDH06.

1.7 Jiulongshan, Ningxia, China

Jiulongshan cemetery is located on Jiulong Mountain, about 1.3 km southwest of Yuanzhou District, Guyuan City, which now belongs to Yangfang Village in Kaicheng Town. It is named after its shape resembling nine dragons. From April to May in 2009, rescued excavations were carried out due to the theft of the cemetery. A total of 11 caverns were discovered, including a number of important skull specimens of cattle, horses and sheep, and the Spring and Autumn Period cultural relics. More than a hundred animal remains were excavated and the number of goats and sheep are much higher than other animals like horse and cattle (Luo 2018). Two samples from Jiulongshan were used in this study, including JLS05 and JLS06.

1.8 Zhongzhuang, Ningxia, China

Zhongzhuang the Spring and Autumn Period cemetery is located in Zhongzhuang Village, Chengyang Town, Pengyang County. It is about 30 kilometers from Chengyang Town or 33 kilometers from Pengyang County. After investigation, exploration, and excavation of the cemetery from August to September in 2008, a total of two tombs were found. A large number of skulls and hooves of horses, cattle, and sheep were unearthed from the tomb, and a small number of bronzes, bones, and agates were also found. Among different animal remains, goat and sheep are the dominant species (Luo 2018). There was no farm tool on this site. The date of this site is approximately at ~2,500 years ago (Zhao et al. 2010). One sample (ZZ01) from Zhongzhuang was used in this study.

1.9 Tianxi, Henan, China

In May 2015, in order to cooperate with the construction of the Tianxishangcheng project in the eastern part of Gongyi City, the Gongyi City Institute of Cultural Relics and Archaeology excavated a Song cemetery in the area, which was called number 230. This cemetery is boot-shaped, which is a common style of the Song Dynasty. The passage of the cemetery is located in the south of the burial room, 2.06 meters in length, 0.74 ~ 1.14 meters in width and 3.8 meters in depth. The burial room is nearly rectangular, 2.32 meters long and 1.48 meters wide. The burial room is about 0.6 meters below the cemetery's passage. It is flat and straight. The cemetery has been stolen, so the funerary

objects and human bones were lost. But the skeleton of the burial animals was retained, which were identified as 13 goats and one cattle. Seven samples (GTM01, GTM02, GTM03, GTM04, GTM06, GTM08, and GTM11) in Tianxi were used in this study. Among them, three samples were radiocarbon dated to about 450 YBP (Table S2). In this study, we regard all samples from this site as ~450 YBP.

Supplementary Note 2: DNA extraction, library preparation, and sequencing

The dust and clay on the outer surface of teeth or bones were cleaned with a fur brush. Subsequently, the cleaned samples were cut into smaller pieces and soaked in 10% bleach for 20 min, rinsed with ethanol and distilled water, and then UV-irradiated for 30 min on each side. Finally, the samples were powdered under liquid nitrogen in a 6850 Freezer Mill (SPEX CertiPrep, Metuchen, NJ, USA).

Ancient DNA was extracted from the sample powder by using a modified silica-spin column method (Yang et al. 1998) in a dedicated ancient DNA laboratory at Jilin University. The 200 mg powders were incubated overnight with 3 ml of lysis buffer (0.5 M EDTA pH 8.0 and 0.5 mg/ml Proteinase K) in a rotating hybridization oven at 50℃ (220 rpm/min). After centrifugation, the supernatant was transferred into an Amicon® Ultra-4 centrifugal filter device (Merck Millipore Ltd, 10000 Nominal Molecular Weight Limit), reduced to less than 100 ul, and purified with QIAquick® PCR Purification Kit (QIAGEN) according to its manual.

DNA libraries were prepared using NEBNext® Ultra™ DNA Library Prep Kit for Illumina® (New England Biolabs Inc.). And we sequenced all these bar-coded libraries on an Illumina HiSeq X Ten platform (paired-end 150 bp).

Supplementary Note 3: Mapping, filtering and variation calling

The adapter sequences in raw sequencing reads were removed using fastp (S. Chen et al. 2018) (version 0.9.1). To reduce false positives in terms of alignment, we also required the trimmed reads to be at least 15 bp, the number of ‘N’ base less than five, and more than 60% phred base quality values larger than 15. All cleaned reads were aligned against the goat reference genome (ARS1, GCF_001704415.1) (Bickhart et al. 2017) with BWA (Li 2013) (version 0.7.17-r1188). The BWA-backtrack (BWA aln) algorithm (Li and Durbin 2010) (set -l 1024 to disable the seed) was used for ancient samples, and the BWA-MEM algorithm (Li 2013) was used for modern samples. Then, duplicate reads were removed using Picard (version 2.16.0) (https://broadinstitute.github.io/picard/) at the library level. Finally, mapDamage (Jónsson et al. 2013) (version 2.0.8) was applied to characterize the DNA damage pattern and rescale the base quality of ancient samples (**Fig. S2**).

Both GATK HaplotypeCaller (Poplin et al. 2018) (version 3.8) with default parameter and ANGSD (Korneliussen et al. 2014) (version 0.918) (-uniqueOnly 1 -only_proper_pairs 1 -minQ 20 -minMapQ 30 -remove_bads 1 -C 50 -GL 1 -setMinDepth total_mean_depth / 3 -setMaxDepth total_mean_depth * 3) were employed to detect the SNPs in all modern samples. Only biallelic sites detected with both methods with a quality score greater than 50 and no more than 10% missing data were used. To minimize false-positive SNP sites, SNP detection in the ancient samples was restricted to genomic

sites that were confirmed in the modern samples during the last step. Thereafter, all transition sites were also excluded in ancient samples using bcftools (Li et al. 2009) (version 1.6) with parameter -e '(REF="C" & ALT="T") | (REF="T" & ALT="C") | (REF="G" & ALT="A") | (REF="A" & ALT="G")'. For the low-to-moderate coverage samples that had a higher error rate in terms of genotyping, we also used ANGSD to estimate genotype likelihoods in both modern and ancient samples.

Supplementary Note 4: Gender inference

To infer the gender of ancient samples, we calculated the mean depth of SNP in autosomes, X chromosome, and Y chromosome using pysam (https://github.com/pysam-developers/pysam). Since the X chromosome is diploid in the female while it is haploid in the male, the depth of coverage in male shows only half of that in the female. In addition, the Y chromosome in the male is haploid but the female is absent. Given the facts above, we calculated the relative coverage of the X and Y chromosome. Then, a Gaussian Mixture Model was applied to cluster the samples into two clusters using scikit-learn (Pedregosa et al. 2011). The cluster with higher relative coverage in the Y chromosome was identified as male while the other was identified as female (**Figure S23**).

Supplementary Note 5: Sample grouping

We classified all modern samples into six groups according to their geographic location, which include AFR (Morocco, Ethiopia, Nigeria), EUR (France, Netherlands, Italy, Spain, Swiss), SWA (Iran), SAS (Bangladesh, Pakistan), NC (northern China), and SC (southern China). Northern and southern China are divided by the Qinling Mountains-Huaihe River line (Jian et al. 2012). The difference between the north and south sides of the Qinling Mountains-Huaihe River line reflected in various aspects: (1) The south has greater monsoon rainfall, while the north is a warm-temperate arid and semi-arid climate; (2) The south has more red soil, while the north has more yellow/brown soil; (3) The south distributed more evergreen broad-leaved woodland in a subtropical forest, while the north distributed more deciduous broad-leaved forest. Besides, the Qinghai-Tibet Plateau was also classified as northern China due to its cold environment. And, all these published ancient genomes were aggregated in the following analysis according to (Daly et al. 2018), including three Neolithic goat populations around the Fertile Crescent: Neolithic West (Anatolia and the Balkans), Neolithic East (Iran and Turkmenistan) and Neolithic Levant (Jordan and Israel) (**Table S3**).

Supplementary Note 6: Principal component analysis

To include the low coverage sample into the PCA analysis, we employed smartpca implemented in the EIGENSOFT package (Patterson et al. 2006) (version 7.2.1). The principal components were calculated with modern samples, then the ancient samples were projected onto the first three components, using the ‘lsqproject’ and ‘autoshrink’ options (**Fig. 1b, Fig. 2b, Fig. S3**).

Supplementary Note 7: Phylogenetic analysis

To reconstruct a phylogenetic relationship between ancient and global modern samples, a pairwise genetic distance matrix was calculated using plink (Purcell et al. 2007) (version 1.9) from genotype probabilities. Then a neighbor-joining tree was built via MEGA X software (Kumar et al. 2018) with 100 bootstraps. The topological structure was visualized using iTOL (Letunic and Bork 2016). Only high-coverage samples (>3×) were used in this analysis.

Supplementary Note 8: Mitochondrial DNA analysis

To construct the mitochondrial sequence for each individual, we aligned reads to reference mitochondrial sequence, then used MIA (mapping-iterative-assembler) (Briggs et al. 2009) to assemble the complete mitochondrial genomes. We also retrieved additional whole mtDNA sequences with known haplogroup from GenBank. After multiple sequence alignment using MUSCLE (Edgar 2004) (v3.8.31), we identified a total of 133 diagnostic SNPs showing F_{ST} = 1 between different haplogroups in modern samples and identified haplogroups of each ancient Chinese goat using these sites (Fig. S24).

Supplementary Note 9: *f*-statistic and *D*-statistic analysis

We used AdmixTools (Patterson et al. 2012) (version 4.1) to calculate outgroup *f*3 statistics with Qazvin Bezoar used as outgroup. We observed modern SC exhibited more allele sharing with ancient Chinese goats (**Fig. 2e**, **Fig. S13-14**). The *D* statistics were calculated using Angsd (Soraggi et al. 2018), and argali (*Ovis ammon*) was used as the outgroup (-doAbbababa2 1, -remove_bads 1, -only_proper_pairs 0, -rmTrans 1, -doCounts 1, -minQ 20, -minMapQ 25). The standard errors were estimated through a jackknife method. For all these analyses, the transitions sites were removed to reduce the effect of residual DNA damage on calculations.

Supplementary Note 10: fineSTRUCTURE analysis

We used fineSTRUCTURE v4 (Lawson et al. 2012) to infer the population structure of modern Chinese goats. The phased SNP data was used to produce input PHASE file via plink2chromopainter.pl script. Then, the recombination map was generated using makeuniformercfile.pl script. We run 1,000,000 Burn-in iterations and 1,000,000 sample iterations in the Markov Chain Monte Carlo (MCMC) process. Full Hill Climb tree was built using 10,000 iterations.

Supplementary Note 11: TreeMix analysis

To confirm the population structure of modern Chinese goats, we performed TreeMix analysis using TreeMix v1.13 (Pickrell and Pritchard 2012) (-k 1000, -global), allowing for up to six migration events (-m 0-6). Samples from the same location were grouped (**Table S5**). The inferred trees and residuals were visualized using built-in R script (**Fig. S8**).

Supplementary Note 12: Demographic history analyses

To inferred a detailed demographic history of Chinese Goats, we analyzed the joint frequency spectrums with ∂a∂i (Diffusion Approximation for Demographic Inference) (Gutenkunst et al. 2009). We selected only genomic regions that at least 10 kbp away from any gene locus and excluded the low-alignability regions identified by Genome STRiP2.0 to minimize the effect of selection sweeps. Finally, 233,115 regions with a total length of 454 Mbp were used to infer population history. The two-dimensional site frequency spectra (SFS) of 11 NC samples (NC01, NC02, NC03, NC04, NC06, NC07, NC08, NC09, NC11, NC12, NC13) and 11 SC samples (SC01, SC02, SC03, SC04, SC06, SC07, SC08, SC10, SC11, SC13, SC16) was estimated using Angsd. The folded SFS was used to minimize potential biases. We use a simple model to fit first and increase the complexity of the model gradually. We selected the best model according to the likelihoods and Akaike information criterion. The confidence interval for each parameter was determined by then performing nonparametric bootstrapping (100 times). In this study, the generation time was set at two years and the mutation rate was considered to be 4.32×10^{-9} per base per generation (Zheng et al. 2020).

To compare different demographic scenarios with or without gene flow from EUR to NC (**Fig. S16**), we used an Approximate Bayesian Computation (ABC) approach implemented in DIYABC (Cornuet et al. 2014). Three populations including EUR (FRCH05, ITCH01, ITCH02, ITCH03, ITCH04, ITCH05, FRCH01, FRCH02, FRCH03, FRCH04, and NLCH03), NC and SC (using same samples as in ∂a∂i analysis) were used in this analysis. We performed four million simulations for each scenario using 6,497 SNPs which thinned using a distance filter of interval >50-kb and a rare SNP filter of MAF >0.05. All one-sample summary statistics and two-sample summary statistics were used.

Supplementary Note 13: Genome-wide selection analysis

13.1 Genome-wide selection scan

To detect the selective sweeps in NC and in SC, vcftools (Danecek et al. 2011) (version 0.1.14) was used to calculated F_{ST}, θ_π, and Tajima's D with a 50-kb sliding window and 10-kb step size. The windows with F_{ST} value beyond the three-fold IQR (interquartile range) were classified as outliers. Then, the F_{ST} outlier windows with top 5% quantile ln(θ_π_SC / θ_π_NC) and bottom 5% $\theta\pi$ in NC were classified as NC-selected. And the F_{ST} outlier windows with bottom 5% quantile ln(θ_π_SC / θ_π_NC) and bottom 5% $\theta\pi$ in SC were classified as SC-selected. (**Fig. S17**). For the possible bias in the X chromosome, we identified outliers in autosomes and the X chromosome separately.

13.2: Multi-sequence alignment

The deletion sequence downstream *FGF5* gene (chromosome 6: 95,454,685-95,455,188 bp) was aligned to the human genome (hg19) by the online BLAT tool (Kent 2002) of the UCSC Genome Browser (Kent et al. 2002). Then human genomic coordinate anchored

by the goat sequence were used to extract the phastCons conserved elements across 100 vertebrate species, the H3K4Me1 peak of NHEK cells and verified transcription factor-binding sites from ENCODE data (Rosenbloom et al. 2012; Casper et al. 2017). We converted these files to goat genomic coordinates through liftOver. The inter-species alignment was performed via muscle (version 3.8.31) (Edgar 2004) with sequences of ibex (GCA_003182615.2), sheep (GCF_000298735.2), cattle (GCF_002263795.1), yaks (GCF_000298355.1), camels (GCF_000767585.1), and humans (GCF_000001405.38). This deletion sequence was verified to be non-existent in three Siberian ibex using genomic resequencing data. The phylogenetic tree of these species was from the TimeTree database (http://www.timetree.org) (Kumar et al. 2017).

13.3: Haplotype network analysis

We constructed a haplotype network of *EDA2R* selected region (NW_017189516.1: 17,915,001-18,539,000 bp) using pegas (Paradis 2010) (version 0.11) with 719 highly divergent SNPs between modern NC and SC. Besides all modern goats, only ancient goats with high coverage (>3×) were used, including three in NC (YJL01, YJL02, WDH06), four in Neolithic West (blagotin1, blagotin2, blagotin3, blagotin16), and four in Neolithic East (Semnan1-2, Semnan3, Semnan7, Semnan9). The imputation and phasing were performed using BEAGLE (Browning and Browning 2007; Browning and Browning 2016) (version 4.1).

13.4 Genotype of EDA2R in ancient NC

To explore the state of *EDA2R* in different periods of NC, we investigated the genotypes at 719 SNPs in *EDA2R* selective sweep region (NW_017189516.1:17,915,001-18,539,000 bp) with high divergence ($F_{ST} > 0.4$) between modern NC and SC. Although most ancient NC has only a low coverage, the state of this region can be inferred through the limited loci according to their genotype likelihoods. We first estimated the genotype likelihoods for each locus. Then the average likelihoods were calculated to represent the state of the selected region. For female samples, individuals with likelihoods value larger than 0.75 were classified as homozygous for NC type, lower than 0.25 were classified as homozygous for SC type, and between 0.25 and 0.75 were classified as heterozygous. For *EDA2R* in male samples is haploid, 0.5 was used as a threshold to distinguish the NC type (**Fig. S21**).

Supplementary Note 14: historical temperature in China

To study the correlation between temperature changes and allele frequency of NC-type of *EDA2R* in Chinese goats, we referenced the climatic information (Fig. S22) from an highly influential publication entitled “A preliminary study on the climatic fluctuation during the last 5,000 years in China” (Kochen 1973), which was written by Chu Ko-Chen (Zhu Kezhen), one of the founders of Chinese geography and meteorology This paper summarized the historical records of meteorology and phenology in Chinese history (including archaeological evidence from oracle-bones, phenological written material, gazetteer information, and instrumental records) and drew an outline of the major trend of

climate change in the past 5,000 years in China. The paper has been generally used and widely recognized in scientific research and is cited more than three hundred times (Google Scholar).

Supplementary Note 15: the detection of *FGF5* downstream deletion

We used Genome STRiP (Handsaker et al. 2015) with the default parameter to detect the global CNV in modern samples. However, owing to the limitation of the low-coverage samples, we further used clipped reads to verify the deletion positioned downstream of *FGF5* in all ancient goats. We classify an ancient individual with the *FGF5* downstream deletion only when there are reads spanning the breakpoints of the deletion region, with reads clipped along the breakpoint. Furthermore, the absence of deletion of the region in any ancient individual was only concluded when there are at least two reads located within the deletion region. As a result of the above-mentioned requirements, we found nine ancient Chinese goats and 29 ancient goats around the Fertile Crescent that lack this deletion. Moreover, none of the ancient goats could be classified to carry this deletion, according to the above criteria. Notably, all three high-coverage ancient Chinese goats were classified to lack this deletion.

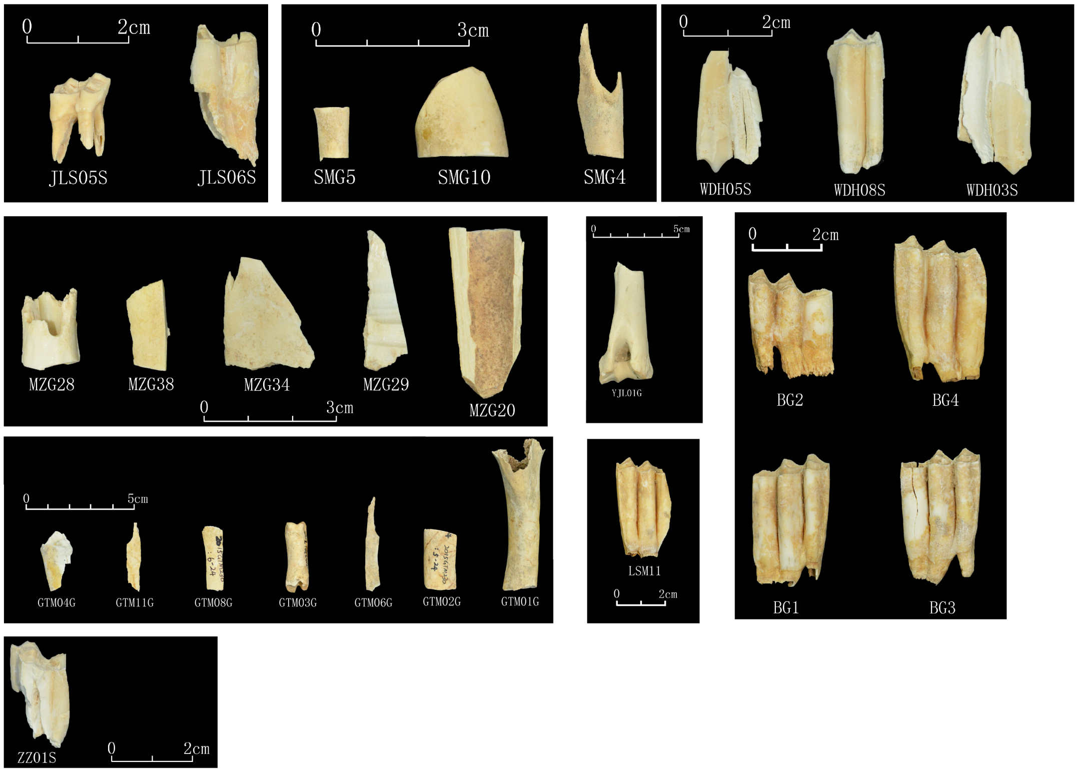

Fig. S1.
Specimens (bones and teeth) of 27 ancient goats excavated in nine sites in this study.

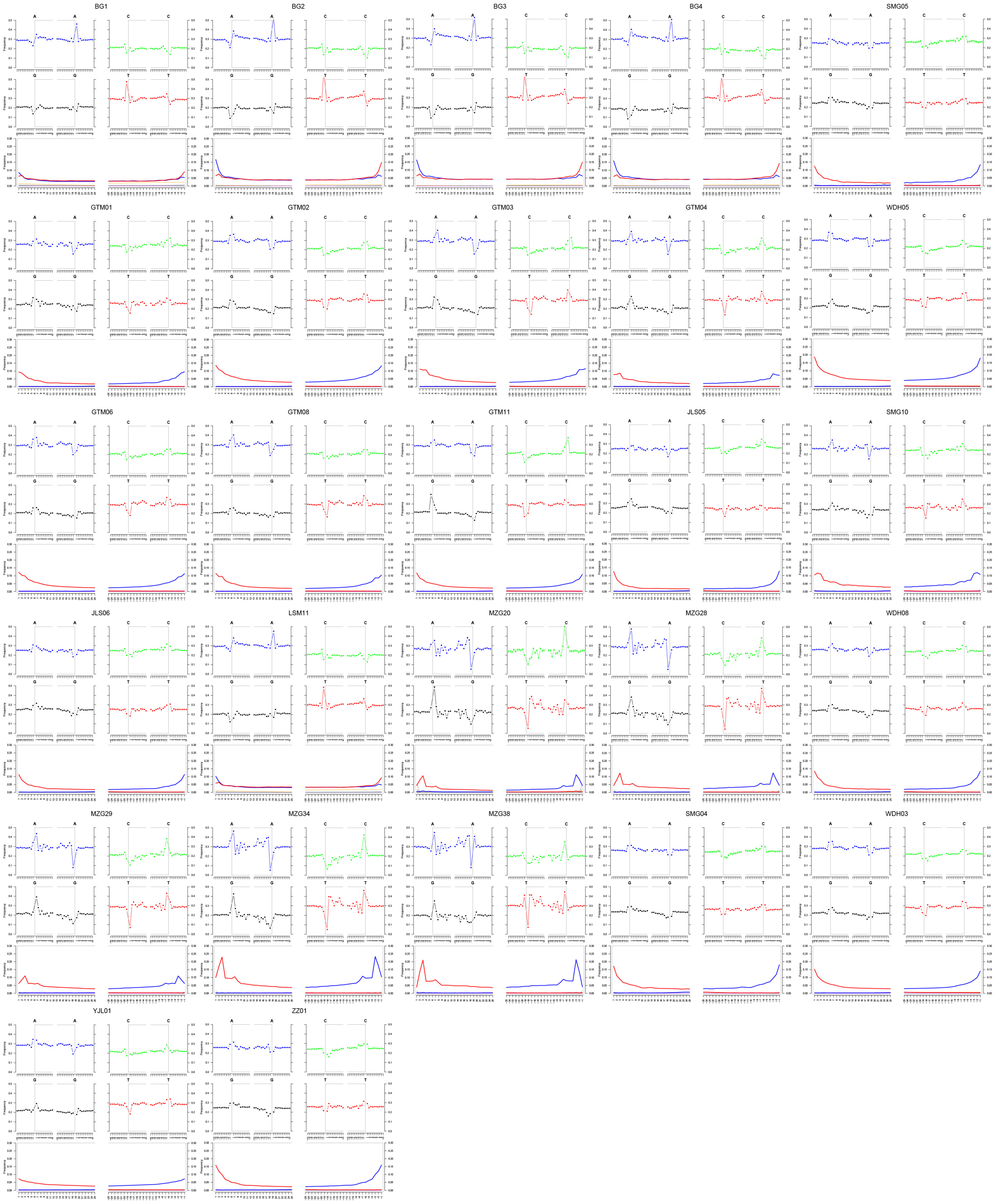

Fig. S2.
Mis-incorporation patterns of 27 ancient samples.

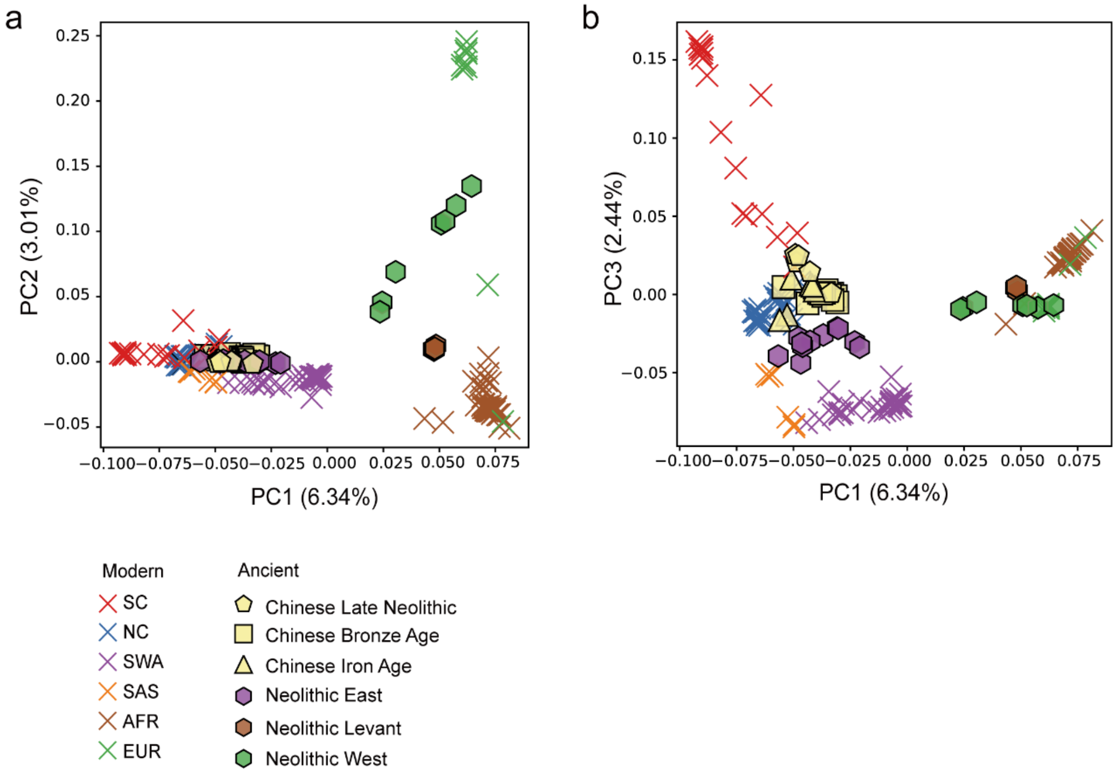

Fig. S3.
Principal component analysis (PCA). All Chinese goats and Neolithic goats around the Fertile Crescent were included. All ancient goats were projected onto the axes computed using modern populations. Modern samples were represented with the cross, while ancient samples with other different symbols according to their periods. The first component was driven by the difference between the East (SWA, SAS, NC, SC) and West (EUR, AFR) population. Then, EUR and AFR were divided in the second component. The third component was mainly driven by the difference within the Asian population.

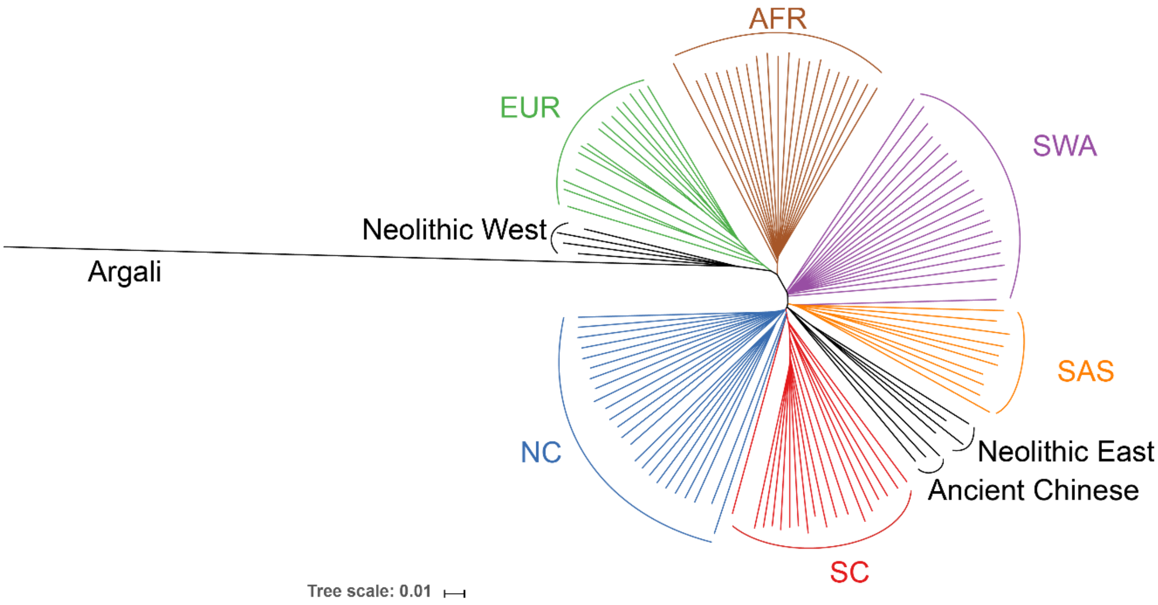

Fig. S4.
Neighbor-joining tree with global modern samples and ancient Chinese samples and Neolithic samples around the Fertile Crescent. Only high-coverage (>3×) samples were included.

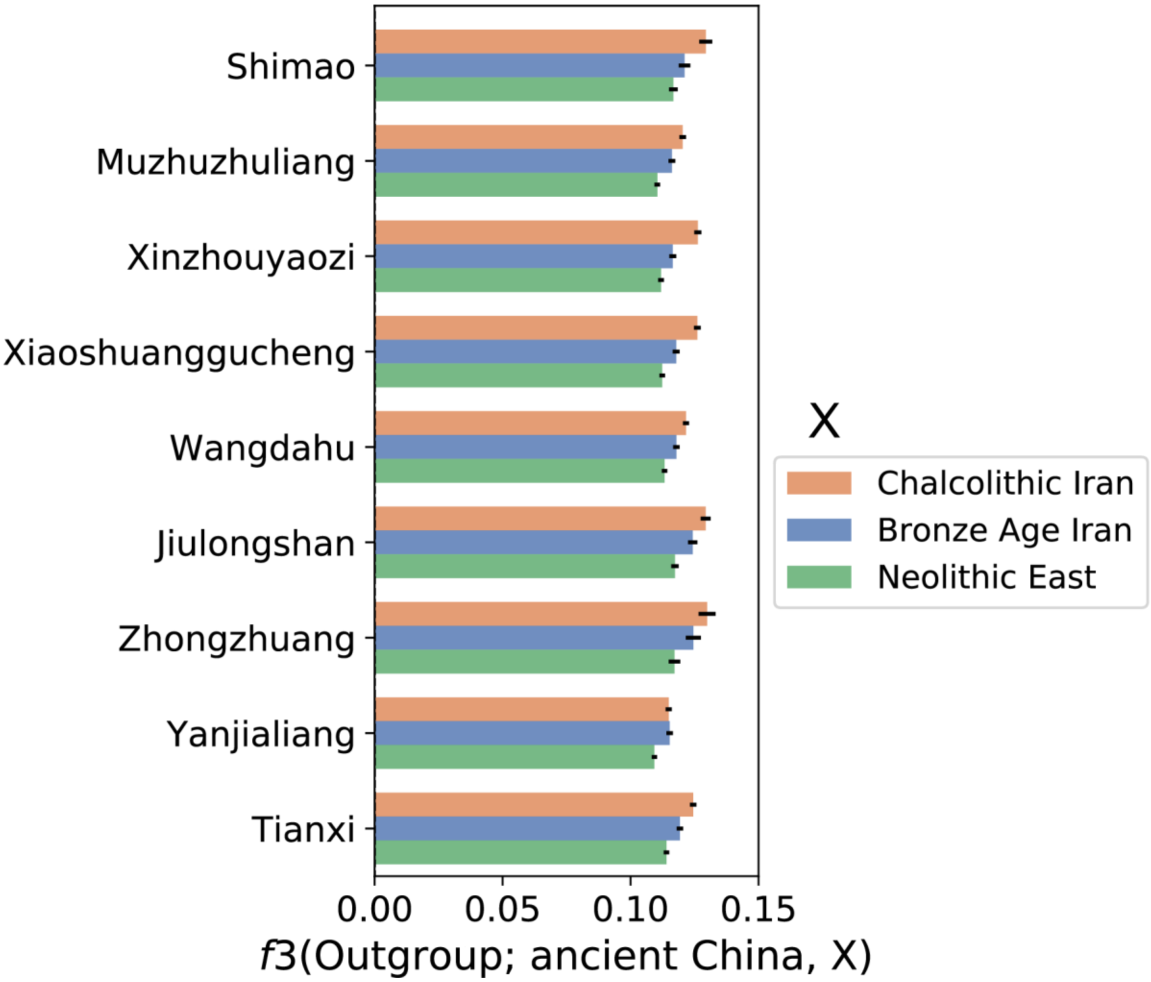

Fig. S5.
Outgroup *f*3 between ancient Chinese goats and ancient eastern Fertile Crescent goats in different periods.

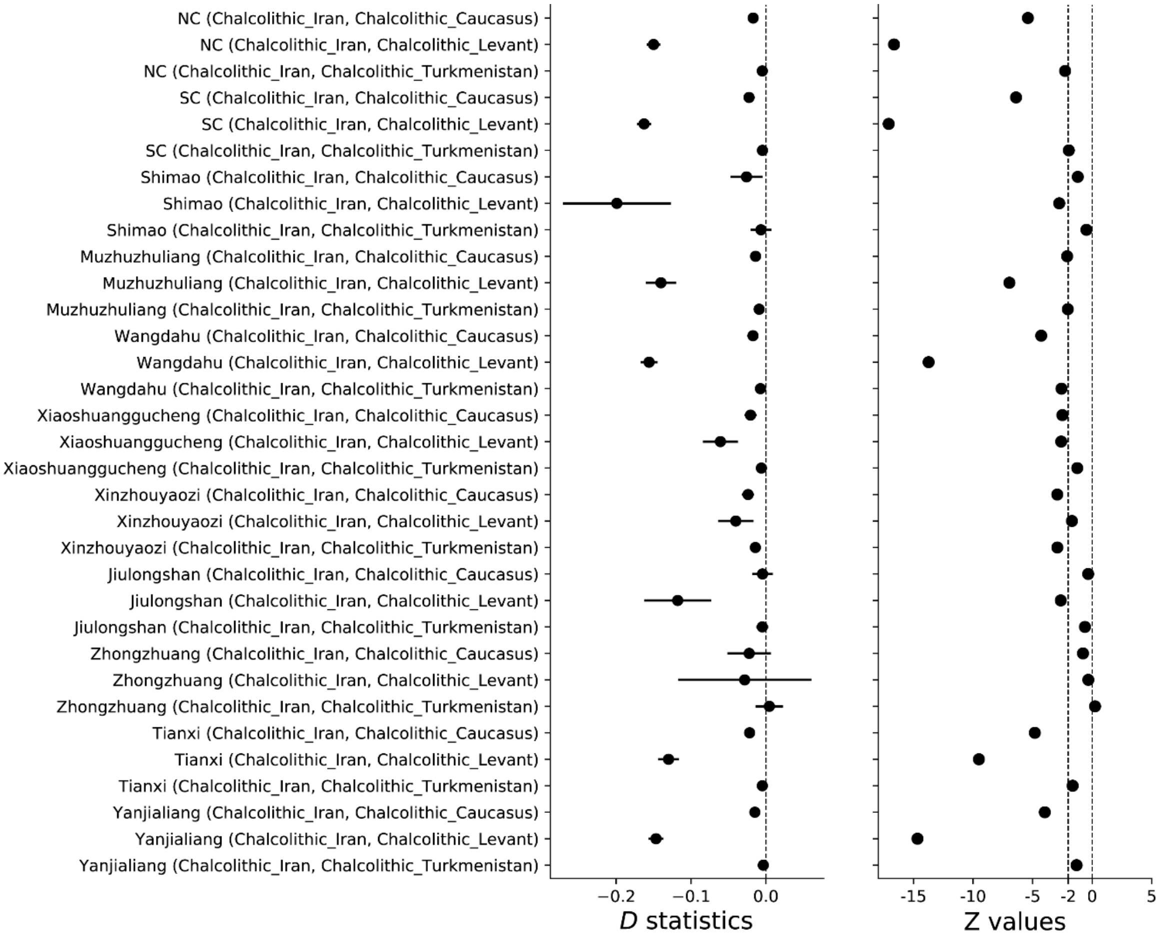

Fig. S6

D statistics to measure the genetic affinity between Chinese goats and Chalcolithic goats in different regions. For each test X (Y, Z), a positive value indicates a higher allele sharing between X and Z, while a negative value indicates a higher allele sharing between X and Z. The result shows the Chalcolithic Iranian goats have the closest genetic affinity with both ancient and modern Chinese goats.

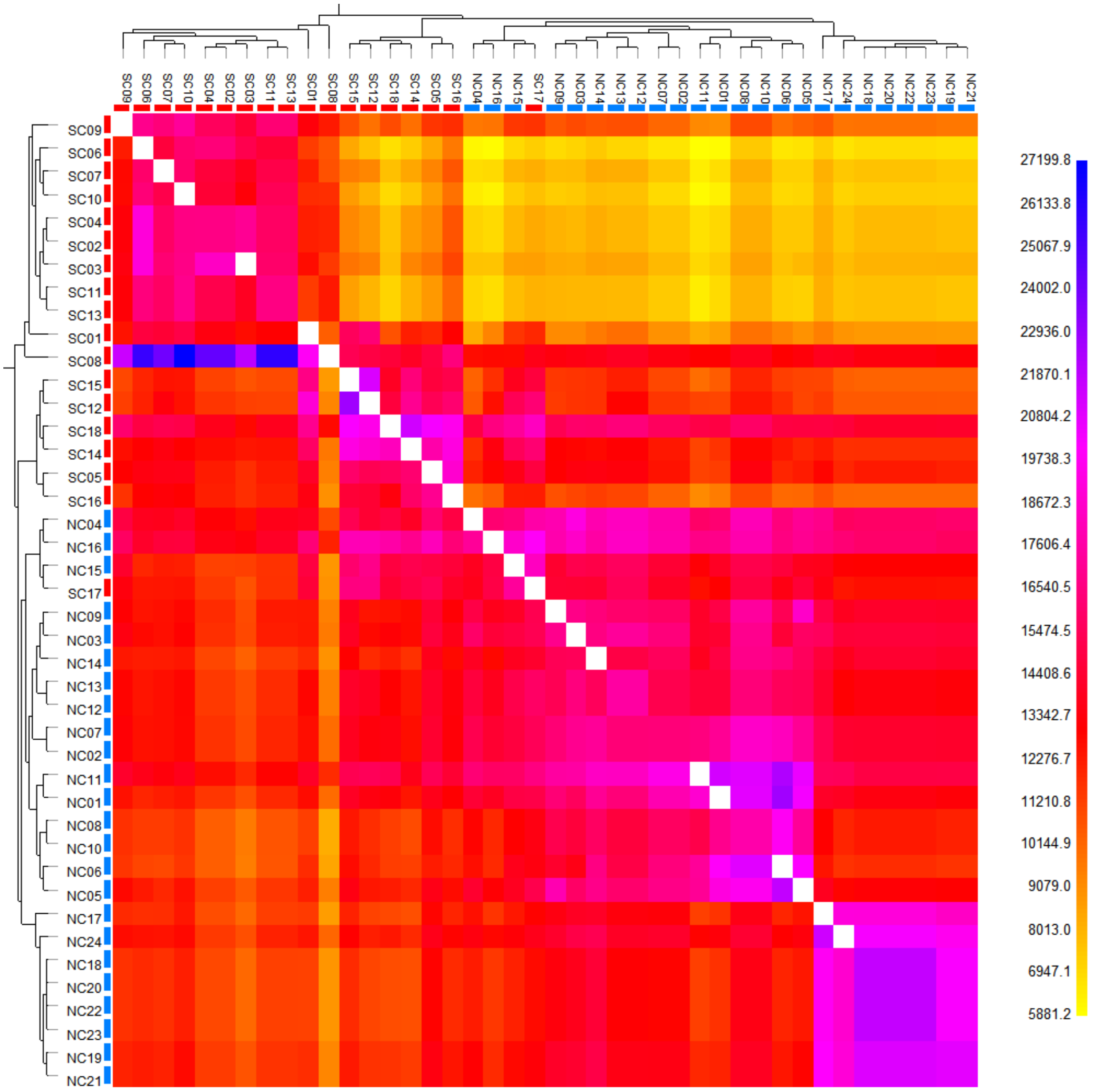

Fig. S7.
Coancestry heatmap for all modern Chinese goats calculated by ChromoPainter/fineSTRUCTURE, which showed the number of discrete "haplotypes" that an individual (rows) receives from other individuals (columns). Adjacent to the heatmap is the dendrogram generated by fineSTRUCTURE, which shows the groupings of the analyzed individuals. The southern Chinese goats are labeled in red, while northern Chinese goats are labeled in blue.

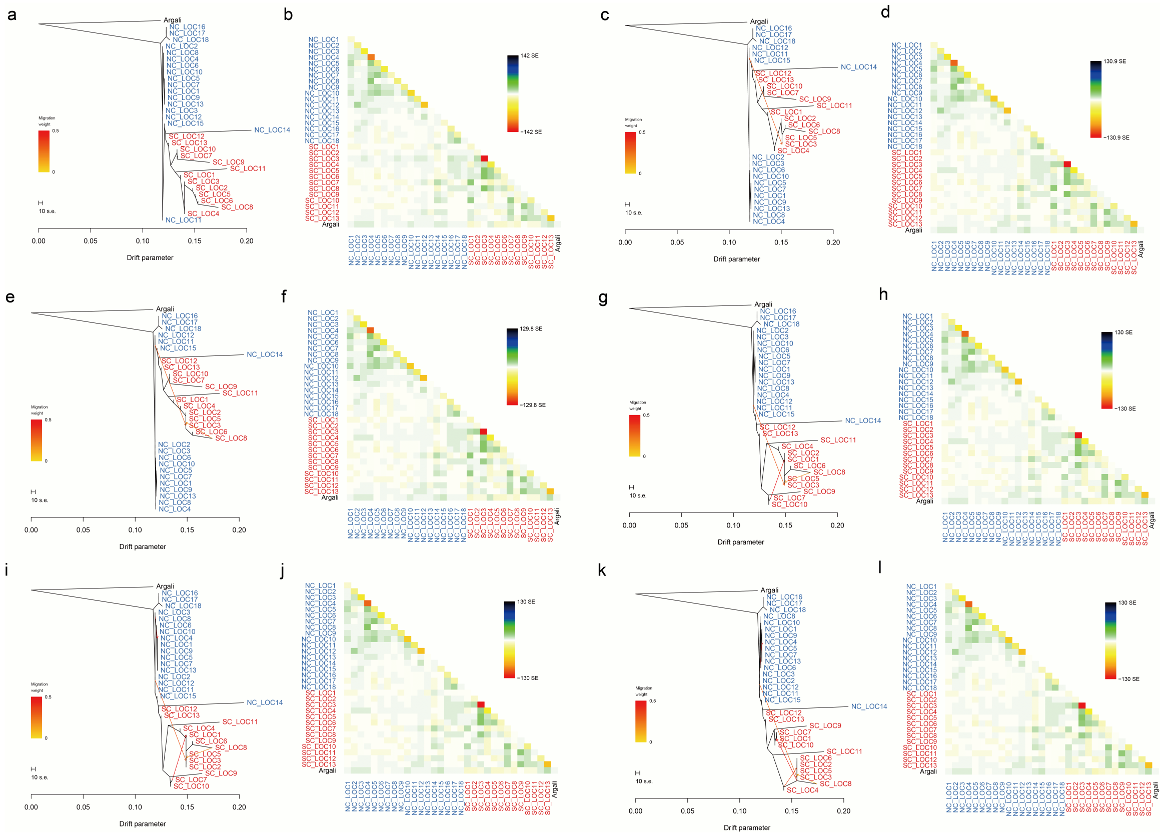

Fig. S8.
TreeMix admixture graphs with zero to six migration edges and the residual fit. All modern Chinese goats were used in this analysis. Samples in identical location were grouped (**Table S5**). The northern Chinese groups were colored in blue, while southern Chinese groups were colored in red.

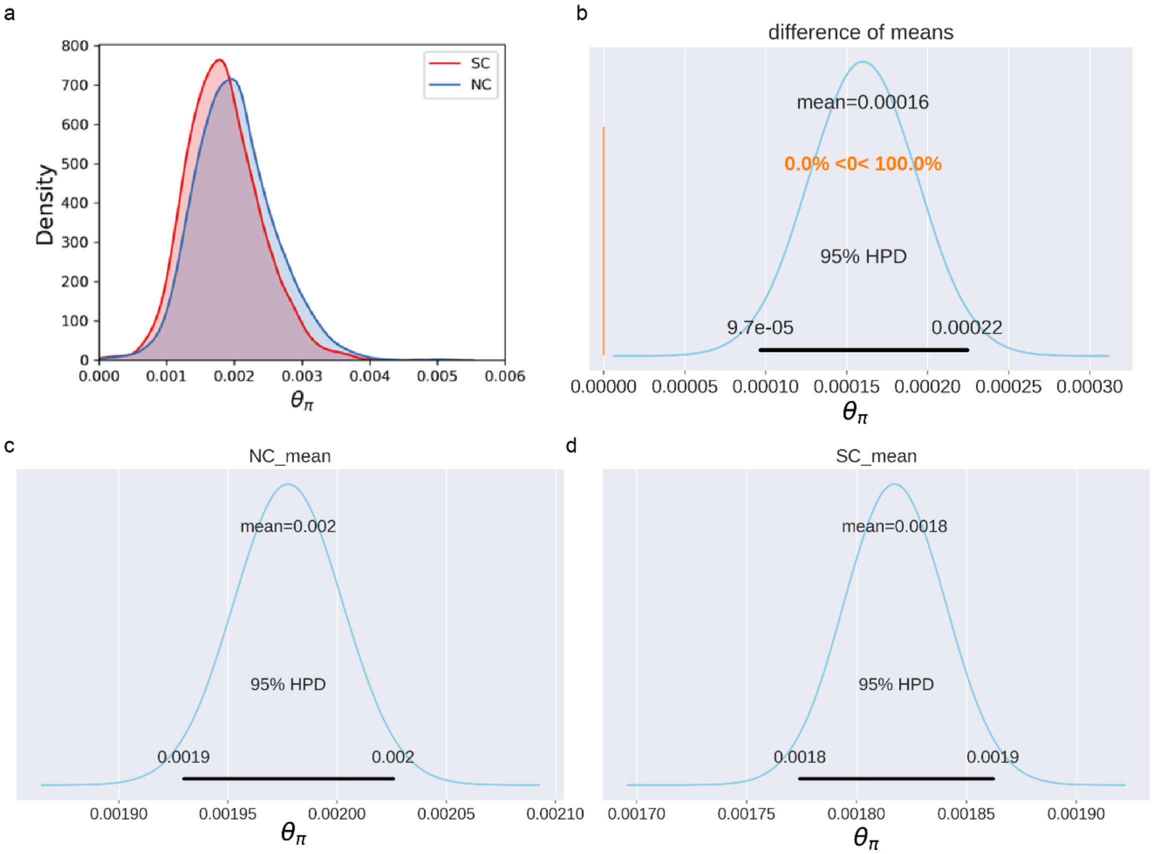

Fig. S9.
(a) The distributions of nucleotide diversity (θ_π) in modern NC and SC (Student's t-test *P* = 2.14 × 10^{24}). The nucleotide diversity was calculated for each group (NC and SC) using 1Mb non-overlapping sliding windows. The posterior distribution of the nucleotide diversity of two groups (**c, d**) and their difference (**b**) were estimated using Bayesian method (Kruschke 2013) via PyMC3 (Salvatier et al. 2016). The 95% HPD (Highest Posterior Density), as a thick black line at the bottom, and the posterior mean are also shown in the plot.

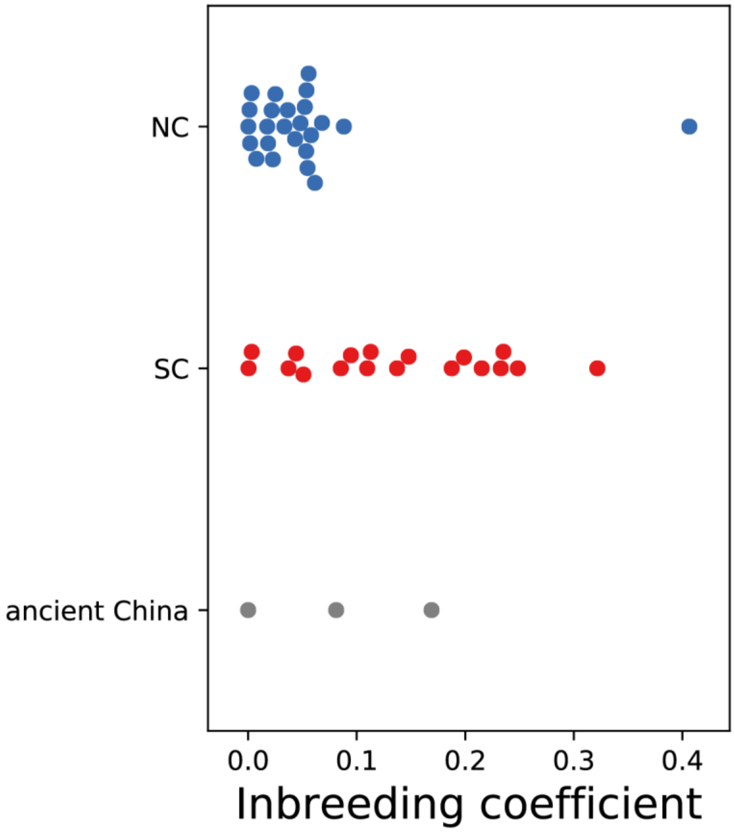

Fig. S10.
Inbreeding coefficient for modern northern Chinese goats (NC), modern southern Chinese goats (SC), and three high-coverage ancient Chinese goats (YJL01, YJL02, WDH06).

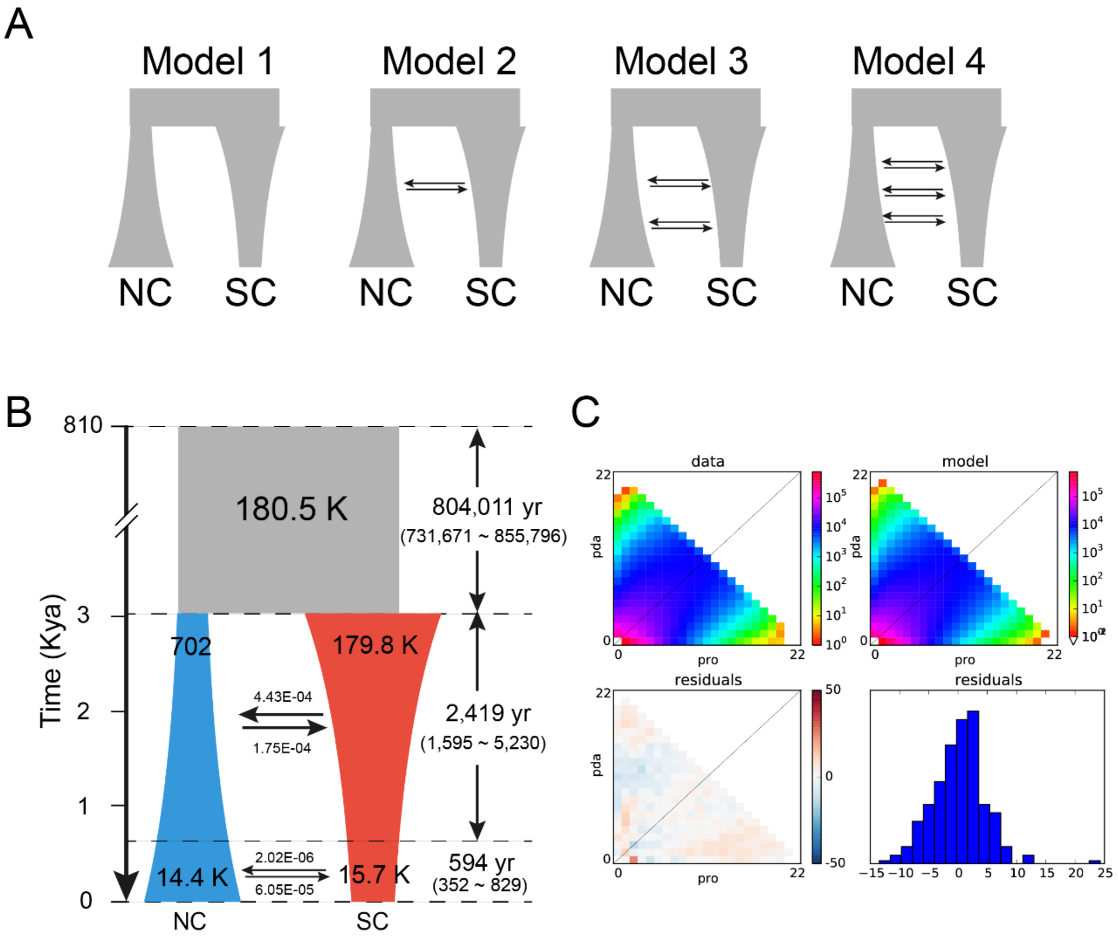

Fig. S11.

Demographic estimation using ∂a∂i. **(a)** Demographic model of northern and southern Chinese (NC and SC) goats using ∂a∂i. In all four models, the ancestral population splits into two (NC and SC), with a fraction *s* going into NC and fraction 1-*s* into SC. Then these two populations begin growing exponentially. In model 1, there is no migration between NC and SC. And in model 2-4, one to three times of asymmetric migration was allowed between them. **(b)** The best-fitted model (model 3) and **(c)** the allele frequency spectra difference between the modeled and real data for the best-fitted model.

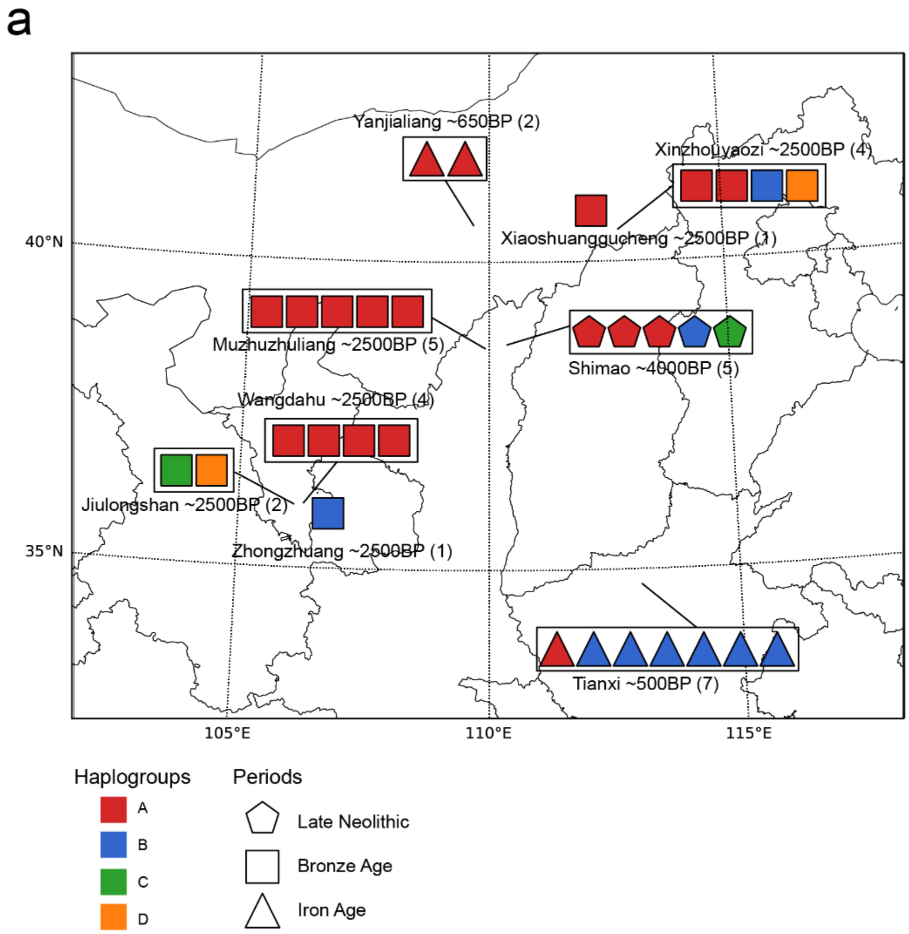

Fig. S12.
Distribution of mitochondrial haplogroups in 31 ancient goats in China.

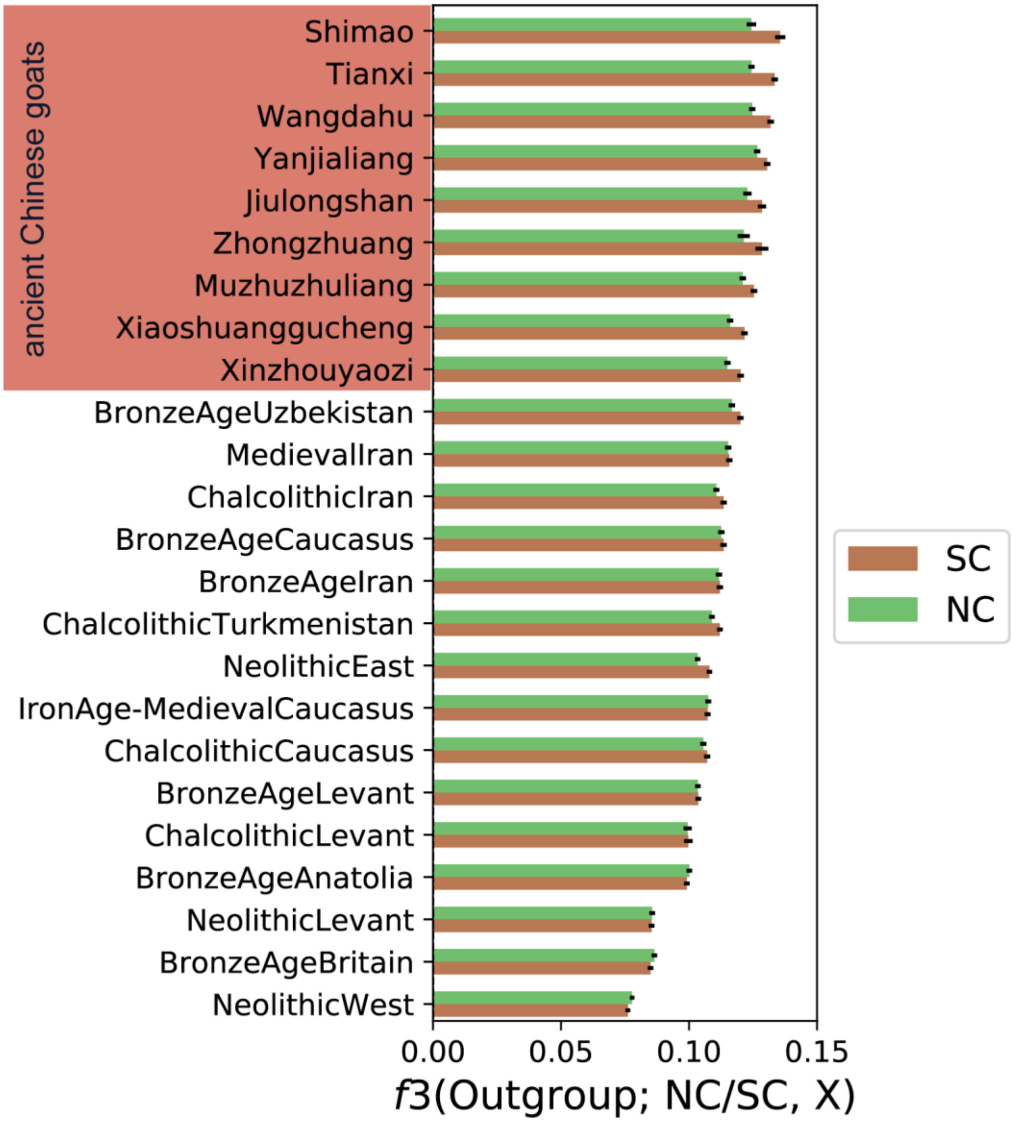

Fig. S13.
Outgroup *f3* measures the allele sharing between modern Chinese goats and global ancient goats. The results show ancient Chinese goats have the closest affinity with modern Chinese goats.

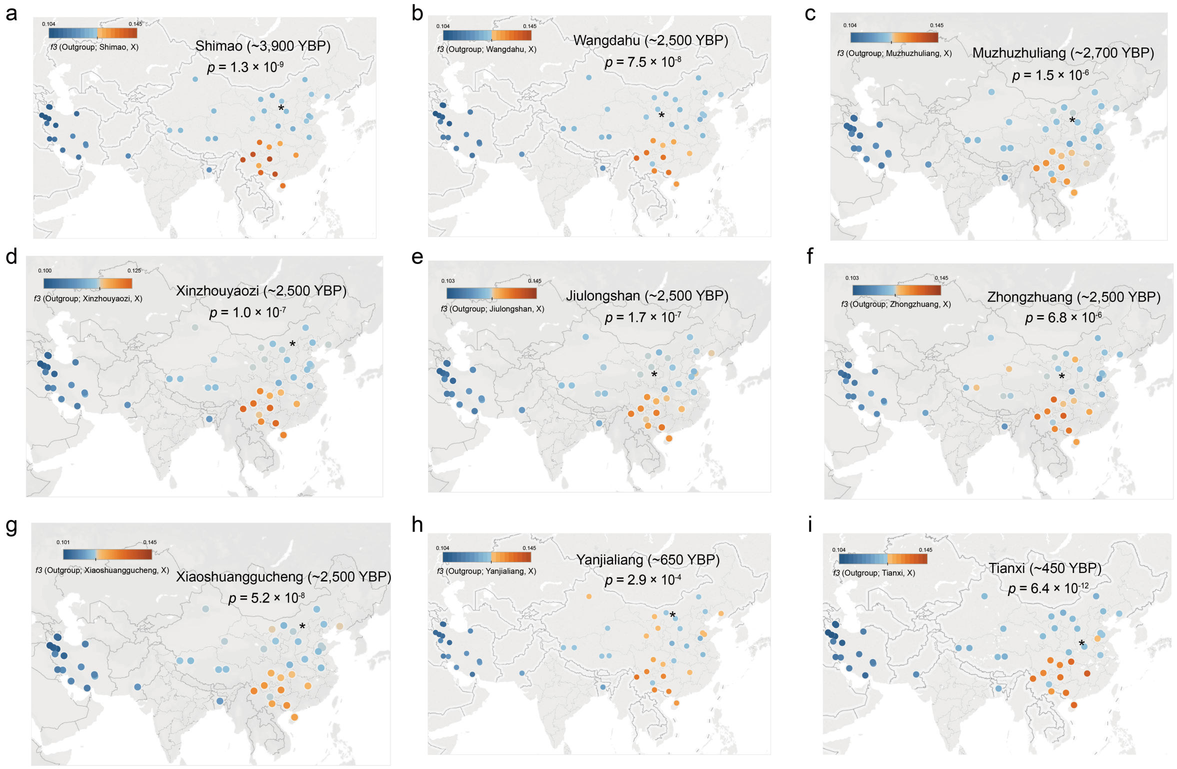

Fig. S14.
The outgroup *f*3 between modern Asian goats and ancient goats in different sites. The position of each archeological site is marked with an asterisk. The Student's t-test was performed in each site to compare outgroup *f*3 between ancient goats and NC/SC. The result show all the ancient Chinese goats have a significant closer affinity with SC than NC (*P* values range from 6.4×10^{-12} to 2.9×10^{-4}).

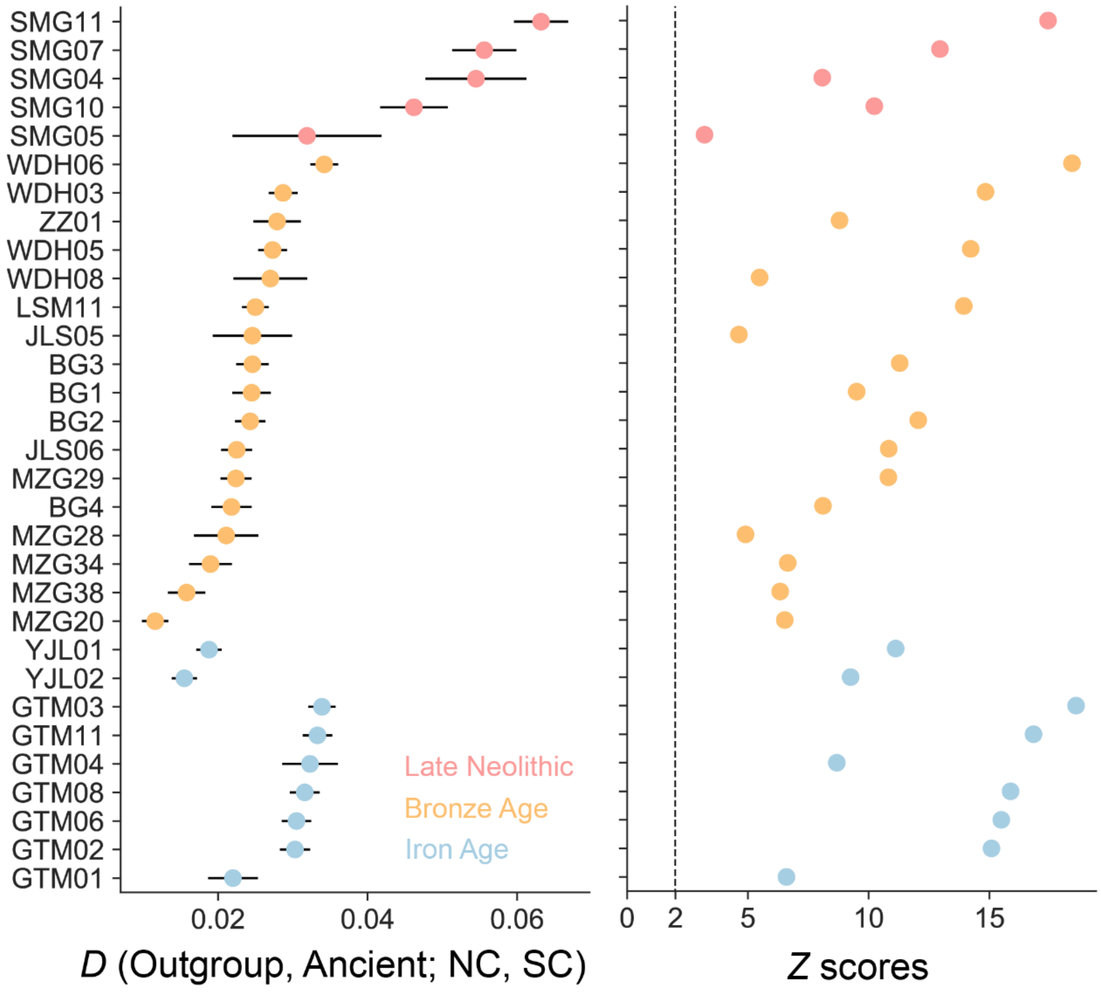

Fig. S15.

D statistics with the form *D* (Outgroup, X; NC, SC), using different ancient Chinese as the test population (X). A positive D value indicated the test population has a closer relationship with modern SC than NC.

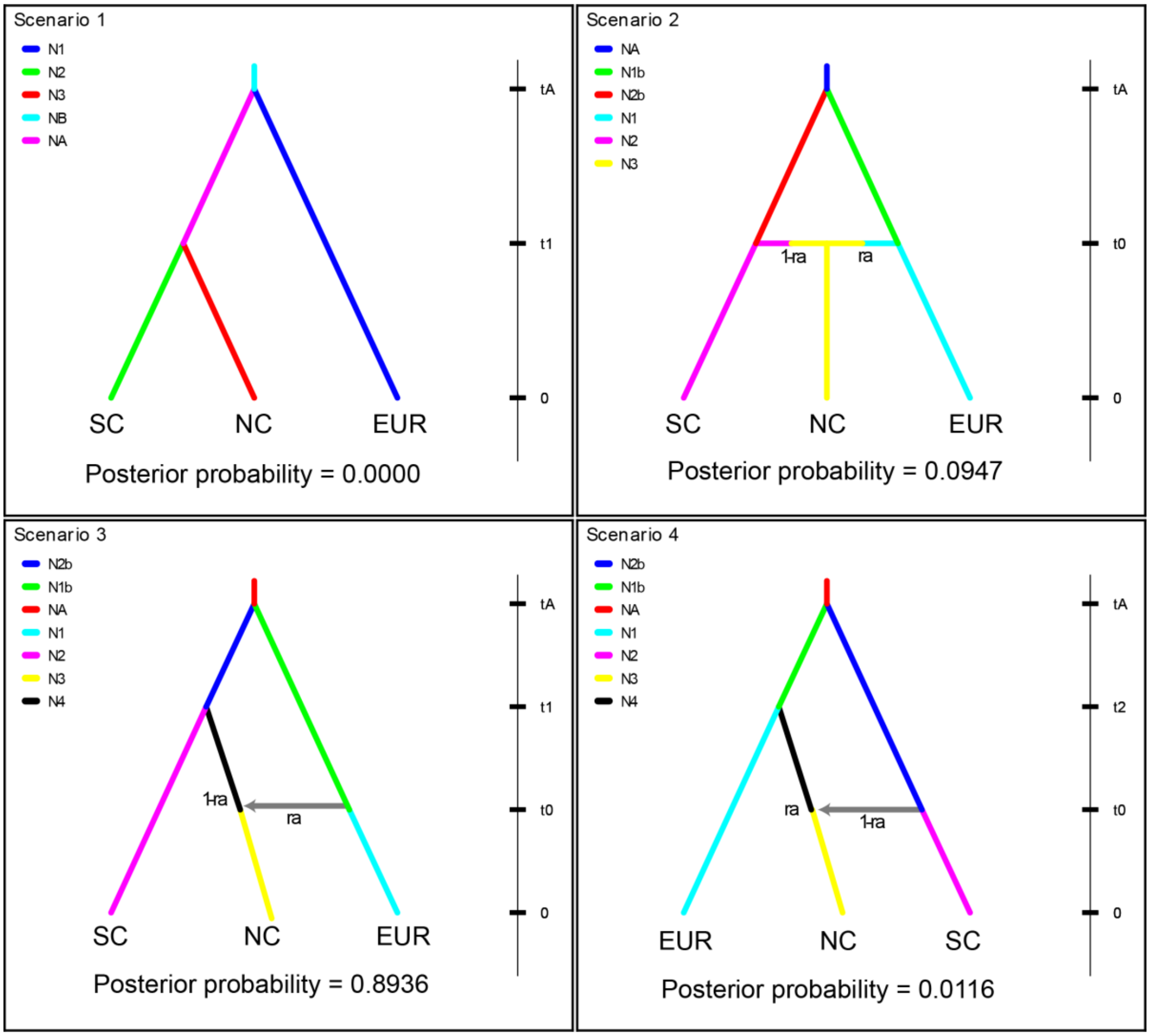

Fig. S16.
ABC models and their corresponding posterior probabilities estimated with logistic regression. Scenario 1 is isolation without gene flow. The ancestral population of Chinese goats and EUR diverged tA generations in the past. Then NC and SC diverged at t1 generation. In scenario 2, there is an admixture event between SC and EUR giving rise to NC. In scenario 3, NC is derived from SC at t1, then EUR introgressed into NC at t0 with the admixture rate ra. Scenario 4 is similar to scenario 3, but NC is derived from EUR and is the source of gene flow from SC. Posterior probabilities of parameters in demographic Model 3 were listed in Table S8.

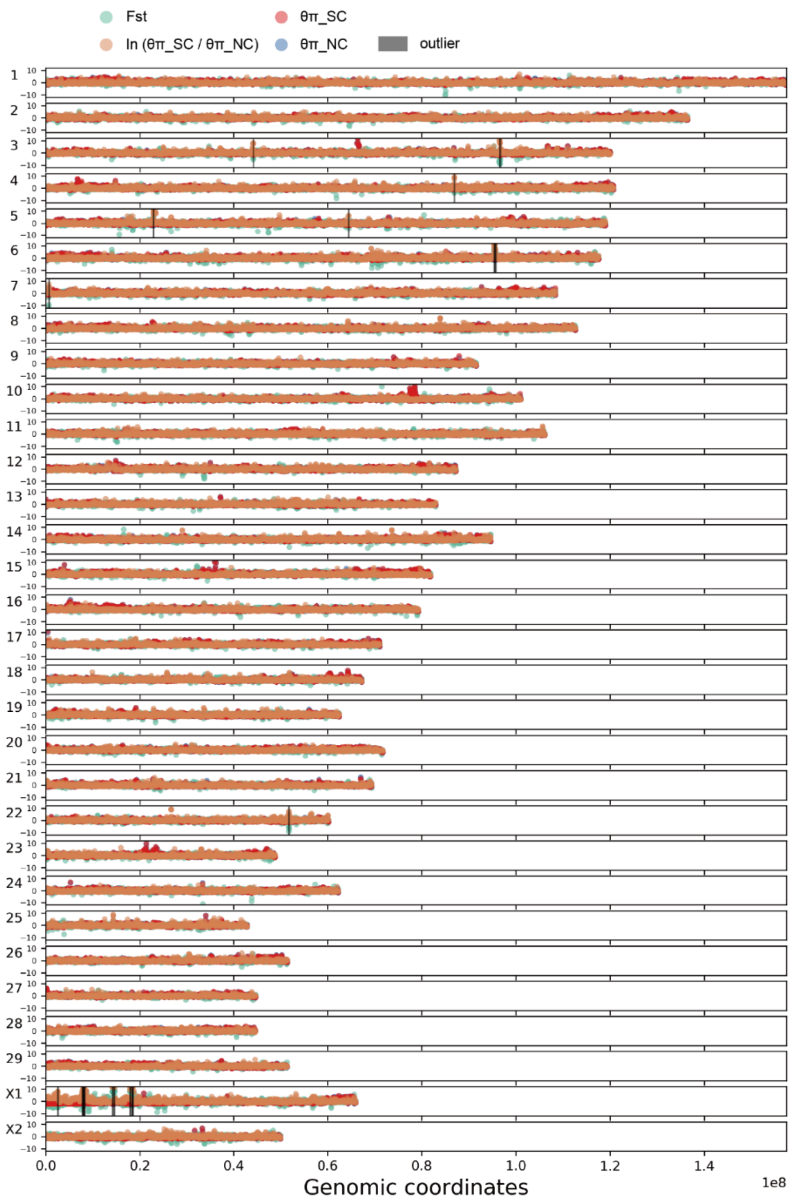

Fig. S17.
F_{ST} and θ_π with 50 kb window size and 10 kb step size in autosomes and X chromosome. The outlier regions were shaded in black.

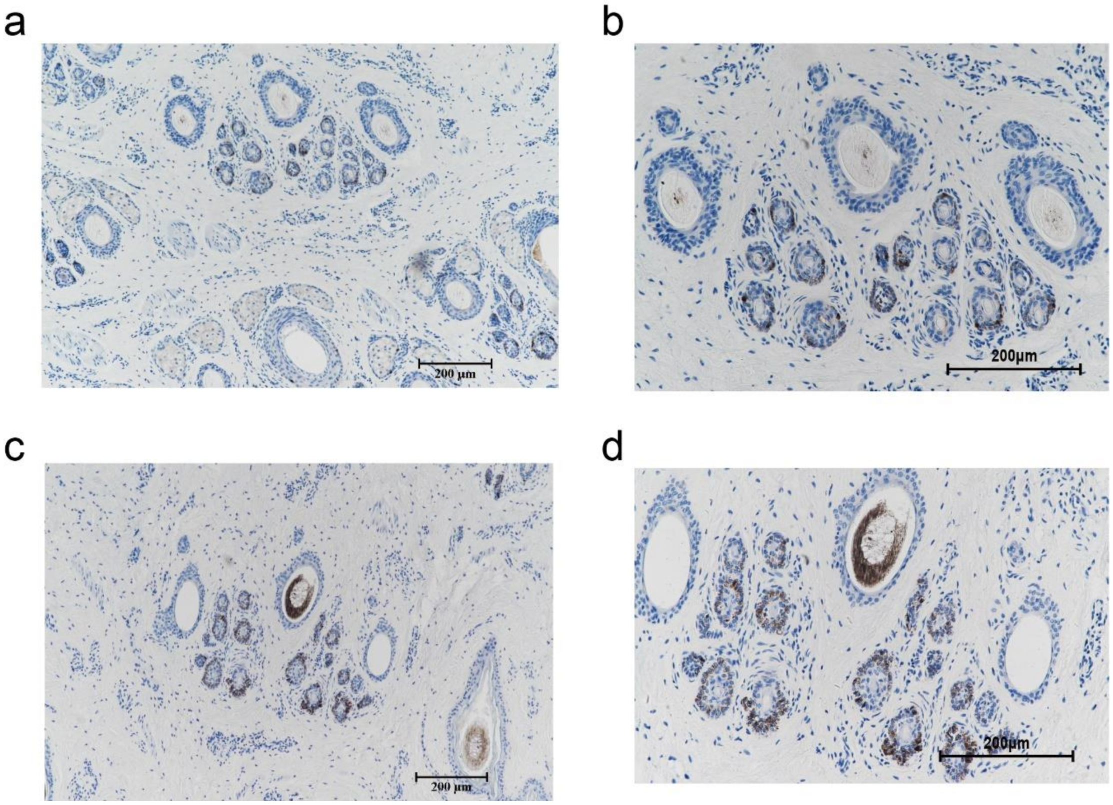

Fig. S18.
Immunohistochemical expression of FGF5 (stained in grey) in skin tissues of northern **(a, b)** and southern **(c, d)** Chinese goats.

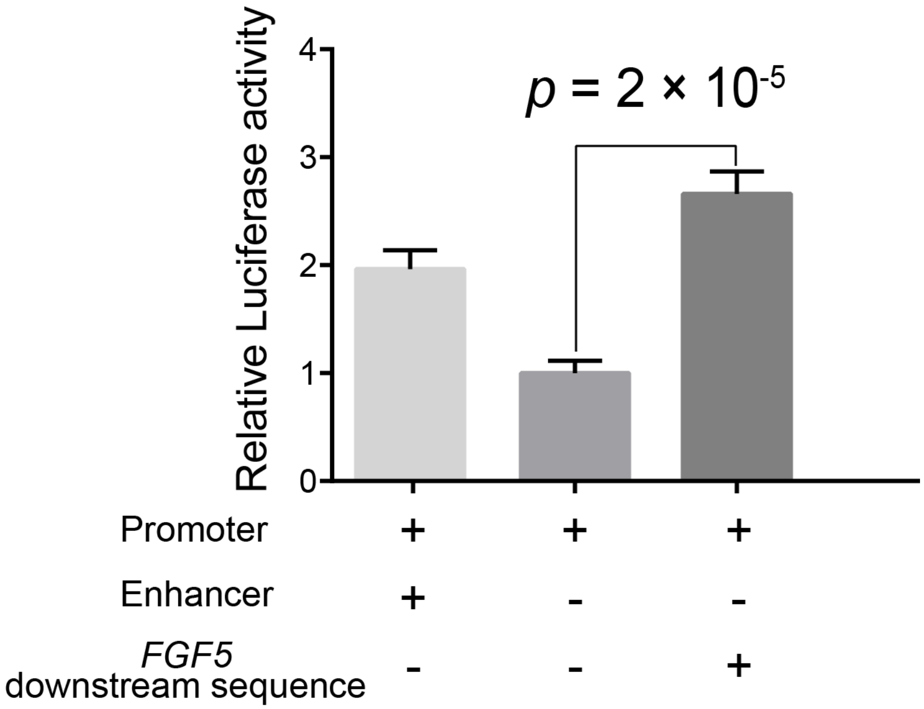

Fig. S19.
The dual-luciferase assay using sheep fibroblasts. The *FGF5* downstream sequence enhanced the activity of Luciferase (Student's t-test $P = 2\times10^{-5}$). Data are shown as mean ± standard error.

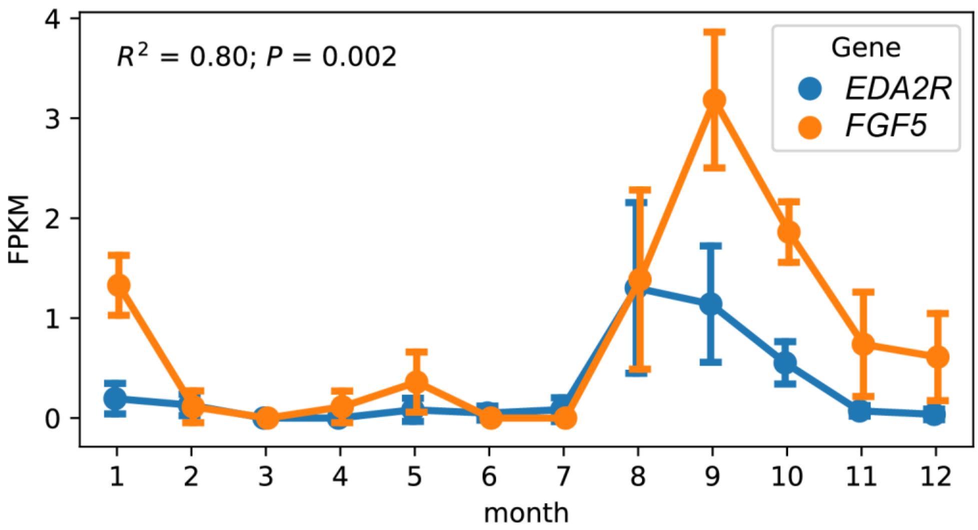

Fig. S20.
The expression of *FGF5* and *EDA2R* in different months of a year. These two genes show similar seasonal pattern (Pearson correlation coefficient = 0.80, P = 0.002). The expression data is downloaded from the NCBI SRA database (PRJNA470971 (Liu et al. 2019)).

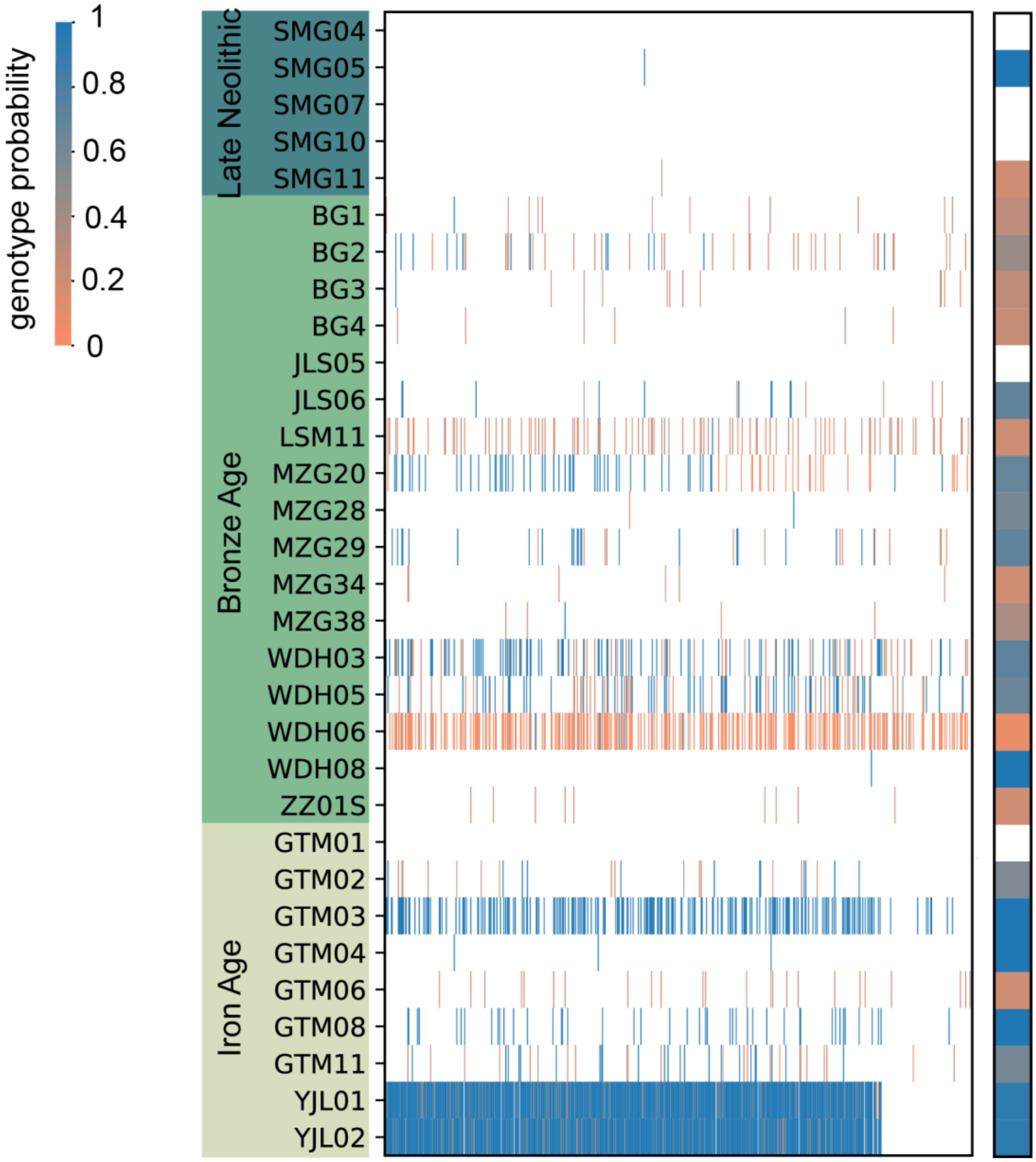

Fig. S21.
Genotype probabilities of the NC-type allele of ancient goats. Sites with no reads covered were shown in white color. The merged genotype probabilities for each individual were shown on the right side. The frequency of NC-type (colored in blue) in the Iron Age is significantly higher than in the Bronze Age.

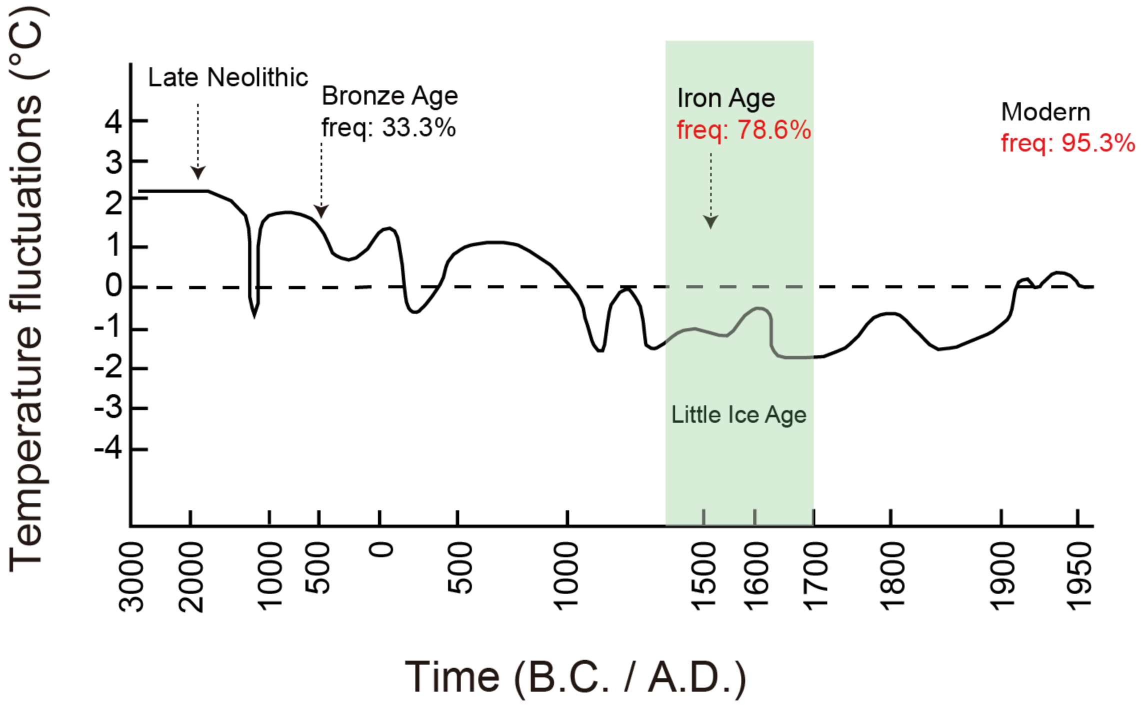

Fig. S22.

Frequency change of selected NC-type of *EDA2R* and temperature fluctuation of China during the last 5,000 years. The frequency of NC-type of *EDA2R* from Bronze Age Chinese goats to modern Chinese goats is mentioned. The frequency in Late Neolithic Chinese goats cannot be accurately assessed due to low coverage. Temperature curve from 3,000 B.C. to the present is studied by (Kochen 1973). The y-axis represents the change of temperature compare to the present temperature level. 0 ℃ temperature line is the temperature level at present.

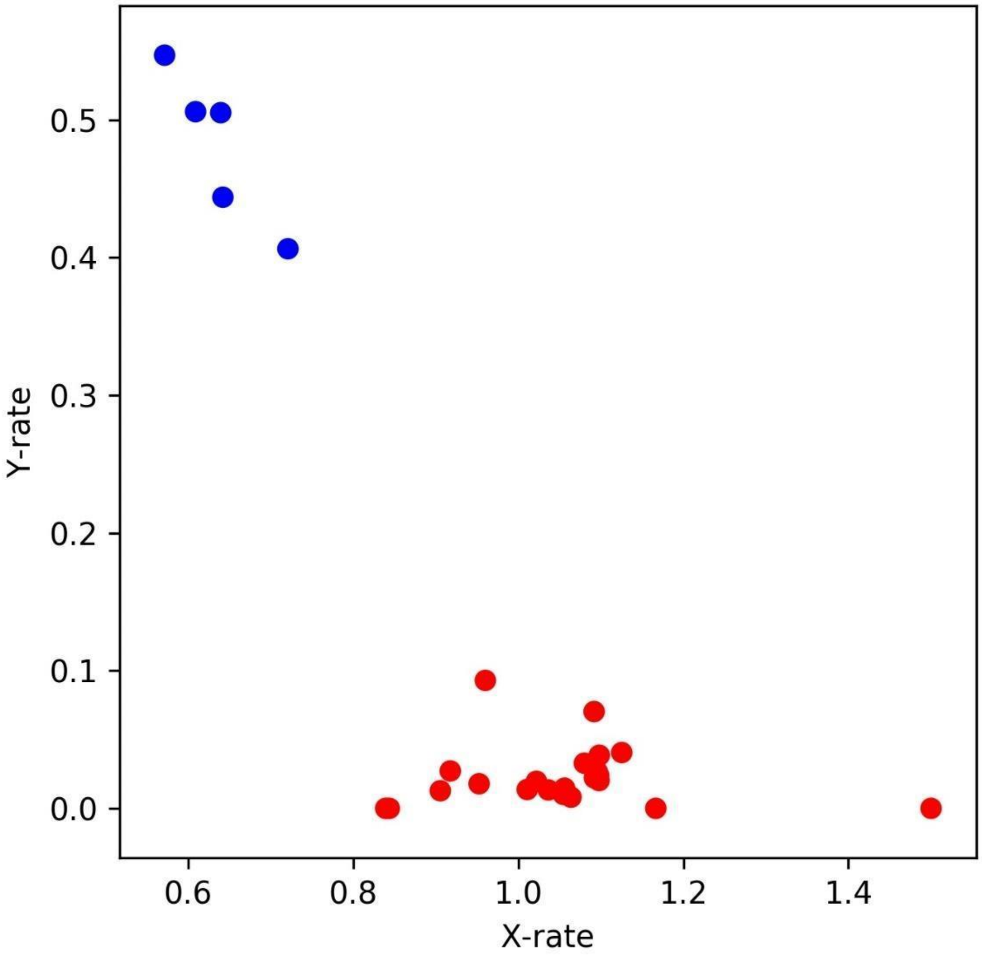

Fig. S23.
Gender inference for ancient samples. Samples colored in blue were identified as males, and those marked with red were identified as females.

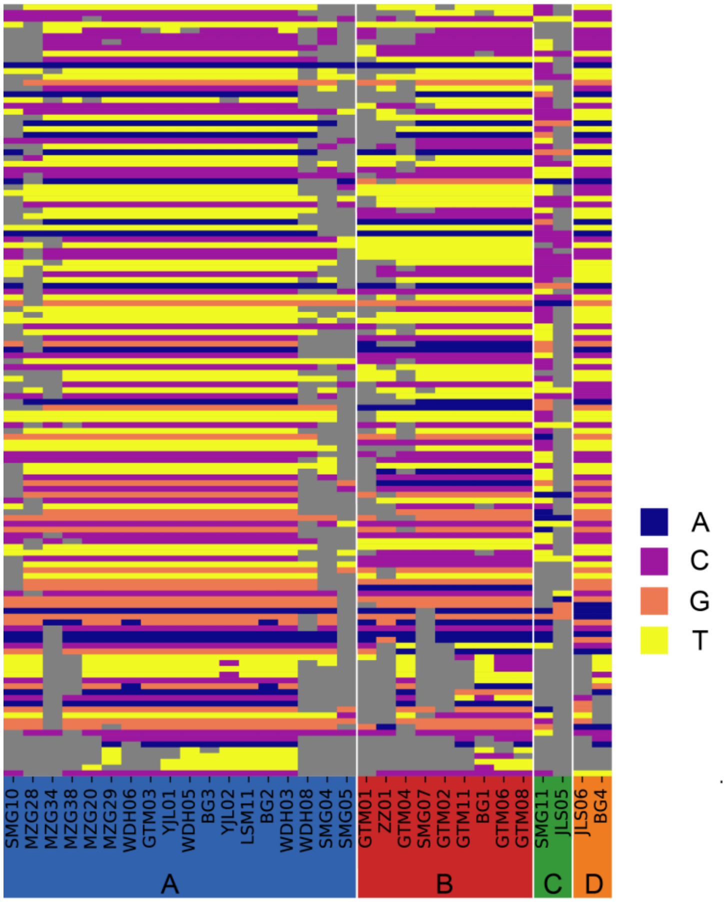

Fig. S24.
Mitochondria haplogroups in all ancient Chinese samples. Missing sites are represented in grey.

Table S1. Overview of ancient sample information and sequencing statistics.

Sample name	Period	Age (years ago)	Location	Sex	Endogenous content	Breadth of coverage	mean depth of coverage
SMG04	Late-Neolithic	~3,900	Shimao	M	0.020	0.012	0.013
SMG05	Late-Neolithic	~3,900	Shimao	F	0.011	0.007	0.007
SMG07*	Late-Neolithic	~3,900	Shimao	M	0.040	0.035	0.036
SMG10	Late-Neolithic	~3,900	Shimao	M	0.118	0.089	0.097
SMG11*	Late-Neolithic	~3,900	Shimao	M	0.016	0.019	0.020
MZG20	Bronze Age	~2,700	Muzhuzhuliang	F	0.397	0.426	1.051
MZG28	Bronze Age	~2,700	Muzhuzhuliang	F	0.031	0.023	0.024
MZG29	Bronze Age	~2,700	Muzhuzhuliang	F	0.181	0.163	0.181
MZG34	Bronze Age	~2,700	Muzhuzhuliang	F	0.157	0.085	0.092
MZG38	Bronze Age	~2,700	Muzhuzhuliang	F	0.162	0.094	0.105
BG1	Bronze Age	~2,500	Xinzhouyaozi	F	0.087	0.062	0.067
BG2	Bronze Age	~2,500	Xinzhouyaozi	F	0.244	0.177	0.204
BG3	Bronze Age	~2,500	Xinzhouyaozi	M	0.136	0.107	0.118
BG4	Bronze Age	~2,500	Xinzhouyaozi	F	0.087	0.056	0.059
LSM11	Bronze Age	~2,500	Xiaoshuanggucheng	F	0.360	0.292	0.366
WDH03	Bronze Age	~2,500	Wangdahu	F	0.853	0.510	0.732
WDH05	Bronze Age	~2,500	Wangdahu	F	0.452	0.431	0.575
WDH06*	Bronze Age	~2,500	Wangdahu	F	0.880	0.978	8.106
WDH08	Bronze Age	~2,500	Wangdahu	M	0.070	0.036	0.038
JLS05	Bronze Age	~2,500	Jiulongshan	F	0.075	0.045	0.047
JLS06	Bronze Age	~2,500	Jiulongshan	F	0.365	0.273	0.354
ZZ01	Bronze Age	~2,500	Zhongzhuang	F	0.190	0.119	0.135
YJL01	Iron Age	~650	Yanjialiang	F	0.772	0.993	7.350
YJL02*	Iron Age	~650	Yanjialiang	M	0.786	0.998	13.439
GTM01	Iron Age	~450	Tianxi	F	0.065	0.047	0.051
GTM02	Iron Age	~450	Tianxi	F	0.088	0.092	0.099
GTM03	Iron Age	~450	Tianxi	F	0.344	0.688	1.524
GTM04	Iron Age	~450	Tianxi	F	0.084	0.017	0.020
GTM06	Iron Age	~450	Tianxi	M	0.282	0.160	0.202
GTM08	Iron Age	~450	Tianxi	F	0.298	0.140	0.181
GTM11	Iron Age	~450	Tianxi	F	0.173	0.166	0.189

NOTE: Samples marked with an asterisk were previously published (Zheng et al. 2020).

Table S2. The radiocarbon age of ancient samples.

Sample	Conventional radiocarbon age	corrected	
		95.4% probability	68.2% probability
YJL02*	650 ± 30 YBP	670 ~ 625 YBP	660 ~ 640 YBP
MZG20	2520 ± 30 YBP	2740 ~ 2680 YBP	2730 ~ 2700 YBP
GTM01	280 ± 30 YBP	438 ~ 350 YBP	428 ~ 378 YBP
GTM02	420 ± 30 YBP	524 ~ 435 YBP	512 ~ 471 YBP
GTM03	330 ± 30 YBP	473 ~ 308 YBP	410 ~ 349 YBP

NOTE: Radiocarbon dating was conducted by Beta Analytic Radiocarbon Dating Laboratory (Miami, FL, USA). "YBP": Years Before Present. YJL02 was previously published (Zheng et al. 2020).

Table S3. Published ancient samples used in this study.

Sample	Region	Period	Group	Bioproject
blagotin1	EUR	Neolithic	Neolithic West	PRJEB26011
blagotin2	EUR	Neolithic	Neolithic West	PRJEB26011
blagotin3	EUR	Neolithic	Neolithic West	PRJEB26011
blagotin16	EUR	Neolithic	Neolithic West	PRJEB26011
AP45	EUR	Neolithic	Neolithic West	PRJEB26011
AP49	EUR	Neolithic	Neolithic West	PRJEB26011
Kov57	EUR	Neolithic	Neolithic West	PRJEB26011
Semnan1-2	SWA	Neolithic	Neolithic East	PRJEB26011
Semnan3	SWA	Neolithic	Neolithic East	PRJEB26011
Semnan7	SWA	Neolithic	Neolithic East	PRJEB26011
Semnan9	SWA	Neolithic	Neolithic East	PRJEB26011
Semnan10	SWA	Neolithic	Neolithic East	PRJEB26011
Semnan13	SWA	Neolithic	Neolithic East	PRJEB26011
Semnan17	SWA	Neolithic	Neolithic East	PRJEB26011
Semnan8	SWA	Neolithic	Neolithic East	PRJEB26011
Fars2-5	SWA	Neolithic	Neolithic East	PRJEB26011
Lur12	SWA	Neolithic	Neolithic East	PRJEB26011
Monjukli8	SWA	Neolithic	Neolithic East	PRJEB26011
Ainghazal1	Levant	Neolithic	Neolithic Levant	PRJEB26011
Ainghazal2	Levant	Neolithic	Neolithic Levant	PRJEB26011
Ainghazal3	Levant	Neolithic	Neolithic Levant	PRJEB26011
Acem1	Anatolia	Bronze Age	BronzeAge Anatolia	PRJEB26011
Acem2	Anatolia	Bronze Age	BronzeAge Anatolia	PRJEB26011
Potterne1	Britain	Bronze Age	BronzeAge Britain	PRJEB26011
Azer3-5	Caucasus	Bronze Age	BronzeAge Caucasus	PRJEB26011
Kohneh2	Caucasus	Bronze Age	BronzeAge Caucasus	PRJEB26011
Tac3	Caucasus	Bronze Age	BronzeAge Caucasus	PRJEB26011
Chalow1	SWA	Bronze Age	BronzeAge Iran	PRJEB26011
Qazvin1	SWA	Bronze Age	BronzeAge Iran	PRJEB26011
Safi2	Levant	Bronze Age	BronzeAge Levant	PRJEB26011
Yarmut1	Levant	Bronze Age	BronzeAge Levant	PRJEB26011
Yarmut7	Levant	Bronze Age	BronzeAge Levant	PRJEB26011
Yoqneam2	Levant	Bronze Age	BronzeAge Levant	PRJEB26011
Bulak1	Uzbekistan	Bronze Age	BronzeAge Uzbekistan	PRJEB26011
Bulak2	Uzbekistan	Bronze Age	BronzeAge Uzbekistan	PRJEB26011
Bulak5	Uzbekistan	Bronze Age	BronzeAge Uzbekistan	PRJEB26011
Azer6	Caucasus	Chalcolithic	Chalcolithic Caucasus	PRJEB26011
Darre1	SWA	Chalcolithic	Chalcolithic Iran	PRJEB26011
Fars1	SWA	Chalcolithic	Chalcolithic Iran	PRJEB26011
Fars4	SWA	Chalcolithic	Chalcolithic Iran	PRJEB26011
Gilat8	Levant	Chalcolithic	Chalcolithic Levant	PRJEB26011
Shiqmim1	Levant	Chalcolithic	Chalcolithic Levant	PRJEB26011
Shiqmim9	Levant	Chalcolithic	Chalcolithic Levant	PRJEB26011
Monjukli1	Turkmenistan	Chalcolithic	Chalcolithic Turkmenistan	PRJEB26011
Monjukli2	Turkmenistan	Chalcolithic	Chalcolithic Turkmenistan	PRJEB26011
Monjukli4	Turkmenistan	Chalcolithic	Chalcolithic Turkmenistan	PRJEB26011
Monjukli6	Turkmenistan	Chalcolithic	Chalcolithic Turkmenistan	PRJEB26011

Azer4	Caucasus	IronAge-Medieval	IronAge-Medieval Caucasus	PRJEB26011
Geor2	Caucasus	IronAge-Medieval	IronAge-Medieval Caucasus	PRJEB26011
Kazbeg1	Caucasus	IronAge-Medieval	IronAge-Medieval Caucasus	PRJEB26011

Table S4. Published modern goats used in this study.

Sample ID	Accession ID	Coverage	gender	Group	Country
ETCH02	SRR5803188	9.74	female	AFR	Ethiopia
ETCH01	SRR5803191	11.03	female	AFR	Ethiopia
MCCH06	ERR229478	13.51	female	AFR	Morocco
MCCH07	ERR229479	12.03	female	AFR	Morocco
MCCH09	ERR229484	15.05	female	AFR	Morocco
MCCH13	ERR234304	12.15	female	AFR	Morocco
MCCH14	ERR234305	13.44	female	AFR	Morocco
MCCH16	ERR246143	12.20	female	AFR	Morocco
MCCH18	ERR246153	14.63	female	AFR	Morocco
MCCH19	ERR248926	13.47	female	AFR	Morocco
MCCH20	ERR248928	14.55	female	AFR	Morocco
MCCH21	ERR248929	13.63	female	AFR	Morocco
MCCH22	ERR248933	13.49	female	AFR	Morocco
MCCH27	ERR313264	15.22	female	AFR	Morocco
MCCH30	ERR315498	13.26	female	AFR	Morocco
MCCH32	ERR315503	15.76	female	AFR	Morocco
MCCH34	ERR315508	14.21	female	AFR	Morocco
MCCH35	ERR315510	12.67	female	AFR	Morocco
MCCH36	ERR315512	12.38	female	AFR	Morocco
MCCH01	ERR219543	12.67	male	AFR	Morocco
MCCH02	ERR219546	13.48	male	AFR	Morocco
MCCH03	ERR229471	13.22	male	AFR	Morocco
MCCH04	ERR229474	12.72	male	AFR	Morocco
MCCH05	ERR229476	13.35	male	AFR	Morocco
MCCH08	ERR229481	13.50	male	AFR	Morocco
MCCH10	ERR229485	12.84	male	AFR	Morocco
MCCH11	ERR229487	13.14	male	AFR	Morocco
MCCH12	ERR232492	12.26	male	AFR	Morocco
MCCH15	ERR234315	13.55	male	AFR	Morocco
MCCH17	ERR246152	13.76	male	AFR	Morocco
MCCH23	ERR299283	13.62	male	AFR	Morocco
MCCH24	ERR313257	12.23	male	AFR	Morocco
MCCH25	ERR313258	13.21	male	AFR	Morocco
MCCH26	ERR313259	15.06	male	AFR	Morocco
MCCH28	ERR313266	12.35	male	AFR	Morocco
MCCH29	ERR313272	13.44	male	AFR	Morocco
MCCH31	ERR315500	13.25	male	AFR	Morocco
MCCH33	ERR315505	13.49	male	AFR	Morocco
MCCH37	ERR315515	11.89	male	AFR	Morocco
MCCH38	ERR315516	14.42	male	AFR	Morocco
MCCH39	ERR315795	11.93	male	AFR	Morocco
MCCH40	ERR318768	12.88	male	AFR	Morocco

MCCH41	ERR332581	11.86	male	AFR	Morocco
MCCH42	ERR332592	11.86	male	AFR	Morocco
MCCH43	ERR340428	12.89	male	AFR	Morocco
MCCH44	ERR345973	11.53	male	AFR	Morocco
NGCH02	SRR5803208	10.84	female	AFR	Nigeria
NGCH01	SRR5803210	8.23	female	AFR	Nigeria
FRCH01	ERR470101	13.45	female	EUR	France
FRCH02	ERR470102	13.25	female	EUR	France
FRCH03	ERR470103	14.23	female	EUR	France
FRCH04	ERR470105	13.89	female	EUR	France
FRCH06	SRR5803174	14.40	male	EUR	France
FRCH05	SRR5803235	13.83	male	EUR	France
ITCH01	ERR405774	14.11	female	EUR	Italy
ITCH02	ERR405775	14.42	female	EUR	Italy
ITCH03	ERR405776	13.69	female	EUR	Italy
ITCH04	ERR405777	13.85	female	EUR	Italy
ITCH05	ERR405778	13.67	female	EUR	Italy
NLCH02	SRR5803227	14.01	female	EUR	Netherlands
NLCH03	SRR5803226	11.11	male	EUR	Netherlands
NLCH01	SRR5803234	13.56	male	EUR	Netherlands
ESCH02	SRR5803173	10.44	female	EUR	Spain
ESCH01	SRR5803232	13.80	female	EUR	Spain
CHCH01	SRR3144625	12.69	female	EUR	Swiss
CHCH02	SRX1560780	11.59	female	EUR	Swiss
BDCH02	SRR5803154	9.21	female	SAS	Bangladesh
BDCH01	SRR5803155	9.27	female	SAS	Bangladesh
BDCH03	SRR5803153	9.91	male	SAS	Bangladesh
PKCH06	SRR5803147	7.76	female	SAS	Pakistan
PKCH04	SRR5803149	12.46	female	SAS	Pakistan
PKCH03	SRR5803150	13.07	female	SAS	Pakistan
PKCH02	SRR5803151	11.53	female	SAS	Pakistan
PKCH01	SRR5803152	13.29	female	SAS	Pakistan
PKCH05	SRR5803148	12.10	male	SAS	Pakistan
IRCH01	ERR297229	12.80	female	SWA	Iran
IRCH02	ERR299449	12.81	female	SWA	Iran
IRCH03	ERR299456	12.63	female	SWA	Iran
IRCH05	ERR313198	11.76	female	SWA	Iran
IRCH07	ERR313200	13.49	female	SWA	Iran
IRCH08	ERR313202	12.85	female	SWA	Iran
IRCH09	ERR313204	12.71	female	SWA	Iran
IRCH11	ERR313207	13.24	female	SWA	Iran
IRCH12	ERR313209	11.78	female	SWA	Iran
IRCH13	ERR313210	12.98	female	SWA	Iran
IRCH15	ERR313212	12.80	female	SWA	Iran
IRCH17	ERR313215	12.41	female	SWA	Iran

IRCH18	ERR340332	11.21	female	SWA	Iran
IRCH21	SRR5602566	13.25	female	SWA	Iran
IRCH37	SRR5803156	9.12	female	SWA	Iran
IRCH34	SRR5803166	7.26	female	SWA	Iran
IRCH36	SRR5803167	10.49	female	SWA	Iran
IRCH32	SRR5803175	13.51	female	SWA	Iran
IRCH39	SRR5803198	9.74	female	SWA	Iran
IRCH38	SRR5803199	6.23	female	SWA	Iran
IRCH30	SRR5803216	9.74	female	SWA	Iran
IRCH31	SRR5803217	10.87	female	SWA	Iran
IRCH26	SRR5803218	11.83	female	SWA	Iran
IRCH29	SRR5803219	16.00	female	SWA	Iran
IRCH27	SRR5803221	15.18	female	SWA	Iran
IRCH28	SRR5803222	12.44	female	SWA	Iran
IRCH23	SRR5803223	11.82	female	SWA	Iran
IRCH24	SRR5803224	10.26	female	SWA	Iran
IRCH25	SRR5803225	8.68	female	SWA	Iran
IRCH04	ERR313197	11.85	male	SWA	Iran
IRCH06	ERR313199	13.44	male	SWA	Iran
IRCH10	ERR313206	11.77	male	SWA	Iran
IRCH14	ERR313211	12.72	male	SWA	Iran
IRCH16	ERR313213	12.88	male	SWA	Iran
IRCH19	ERR340337	10.86	male	SWA	Iran
IRCH20	ERR340339	11.35	male	SWA	Iran
NC01	SRR5803194	9.52	male	NC	China
NC02	SRR5803181	10.63	female	NC	China
NC03	SRR5803197	10.87	female	NC	China
NC04	SRR5803183	9.21	female	NC	China
NC05	SRR5803189	12.80	female	NC	China
NC06	SRR5803180	10.99	female	NC	China
NC07	SRR5803187	10.62	female	NC	China
NC08	SRR5803186	12.99	female	NC	China
NC09	SRR5803182	10.94	female	NC	China
NC10	SRR5803179	12.33	male	NC	China
NC11	SRR5803176	10.09	female	NC	China
NC12	SRR5803206	11.76	male	NC	China
NC13	SRR5803215	13.04	female	NC	China
NC14	SRR5803213	11.23	male	NC	China
NC15	SRR5803177	11.77	female	NC	China
NC16	SRR5803230	8.15	male	NC	China
NC17	SRR5803159	11.84	female	NC	China
NC18	SRR5803157	11.23	female	NC	China
NC19	SRR5803196	9.02	male	NC	China
NC20	SRR5803195	9.33	male	NC	China
NC21	SRR5803203	9.50	male	NC	China

NC22	SRR5803202	10.19	male	NC	China
NC23	SRR5803201	10.05	male	NC	China
NC24	SRR5803160	12.52	female	NC	China
SC01	SRR5803231	12.85	female	SC	China
SC02	SRR5803165	17.75	female	SC	China
SC03	SRR5803162	10.86	female	SC	China
SC04	SRR5803161	13.46	female	SC	China
SC05	SRR5803164	12.15	female	SC	China
SC06	SRR5803163	25.56	female	SC	China
SC07	SRR5803158	18.68	female	SC	China
SC08	SRR5803185	14.85	female	SC	China
SC09	SRR5803184	11.96	female	SC	China
SC10	SRS309478	43.24	female	SC	China
SC11	SRR5803169	12.69	female	SC	China
SC12	SRR5803170	10.66	female	SC	China
SC13	SRR5803233	12.80	female	SC	China
SC14	SRR5803172	8.71	female	SC	China
SC15	SRR5803171	12.86	female	SC	China
SC16	SRR5803228	12.90	female	SC	China
SC17	SRR5803168	12.32	female	SC	China
SC18	SRR5803178	15.77	female	SC	China
IRCA01	ERR340328	6.51	female	bezoar	Iran
IRCA22	IRCA-C3-1002	43.70	female	bezoar	Iran
IRCA02	ERR340329	5.56	male	bezoar	Iran
IRCA03	ERR340330	12.33	male	bezoar	Iran
IRCA04	ERR340331	11.65	male	bezoar	Iran
IRCA05	ERR340333	6.35	male	bezoar	Iran
IRCA06	ERR340334	12.11	male	bezoar	Iran
IRCA07	ERR340335	6.62	male	bezoar	Iran
IRCA08	ERR340336	6.51	male	bezoar	Iran
IRCA09	ERR340338	11.37	male	bezoar	Iran
IRCA10	ERR340340	12.07	male	bezoar	Iran
IRCA11	ERR340341	5.17	male	bezoar	Iran
IRCA12	ERR340342	6.42	male	bezoar	Iran
IRCA13	ERR340343	7.26	male	bezoar	Iran
IRCA14	ERR340344	11.20	male	bezoar	Iran
IRCA15	ERR340345	11.60	male	bezoar	Iran
IRCA16	ERR340347	12.70	male	bezoar	Iran
IRCA17	ERR340348	10.72	male	bezoar	Iran
IRCA18	ERR340426	12.36	male	bezoar	Iran
IRCA19	ERR470100	15.04	male	bezoar	Iran
IRCA20	ERR470104	14.44	male	bezoar	Iran
IRCA21	ERR470106	14.66	male	bezoar	Iran
IRCA23	SRR1576679	16.81	male	bezoar	Iran
IRCA24	SRR5803211	11.56	male	bezoar	Iran

Table S5. Summary of breed information and geographic groups.

SampleID	Accession ID	Breed	Group ID
NC01	SRR5803194	Xinjiang goat	NC_LOC1
NC02	SRR5803181	Xinjiang goat	NC_LOC1
NC03	SRR5803197	Vjimqin White goat	NC_LOC2
NC04	SRR5803183	Chengde Polled goat	NC_LOC3
NC05	SRR5803189	Inner Mongolia Cashmere goat (Erlangshan)	NC_LOC4
NC06	SRR5803180	Liaoning Cashmere goat	NC_LOC5
NC07	SRR5803187	Inner Mongolia Cashmere goat (Alashan)	NC_LOC6
NC08	SRR5803186	Inner Mongolia Cashmere goat (Aerbasi)	NC_LOC7
NC09	SRR5803182	Zhongwei goat	NC_LOC8
NC10	SRR5803179	Chaidamu goat	NC_LOC9
NC11	SRR5803176	Lvliang Black goat	NC_LOC10
NC12	SRR5803206	Yimeng Black goat	NC_LOC11
NC13	SRR5803215	Laiwu Black goat	NC_LOC12
NC14	SRR5803213	Chaidamu goat	NC_LOC13
NC15	SRR5803177	Jining Gray goat	NC_LOC14
NC16	SRR5803230	Yaoshan white goat	NC_LOC15
NC17	SRR5803159	Tibetan goat	NC_LOC16
NC18	SRR5803157	Tibetan goat	NC_LOC17
NC19	SRR5803196	Tibetan goat	NC_LOC18
NC20	SRR5803195	Tibetan goat	NC_LOC18
NC21	SRR5803203	Tibetan goat	NC_LOC18
NC22	SRR5803202	Tibetan goat	NC_LOC18
NC23	SRR5803201	Tibetan goat	NC_LOC18
NC24	SRR5803160	Tibetan goat	NC_LOC18
SC01	SRR5803231	Leizhou goat	SC_LOC1
SC02	SRR5803165	Longlin goat	SC_LOC2
SC03	SRR5803162	Longlin goat	SC_LOC2
SC04	SRR5803161	Longlin goat	SC_LOC2
SC05	SRR5803164	Longlin goat	SC_LOC2
SC06	SRR5803163	Longlin goat	SC_LOC2
SC07	SRR5803158	Longlin goat	SC_LOC2
SC08	SRR5803185	Maguan Poll goat	SC_LOC3
SC09	SRR5803184	Guishan goat	SC_LOC4
SC10	SRS309478	Yunling goat	SC_LOC5
SC11	SRR5803169	Guizhou Black goat	SC_LOC6
SC12	SRR5803170	Xiangdong Black goat	SC_LOC7
SC13	SRR5803233	Jianchang Black goat	SC_LOC8
SC14	SRR5803172	Chuandong white goat	SC_LOC9
SC15	SRR5803171	Matou goat	SC_LOC10
SC16	SRR5803228	Chengdu Brown goat	SC_LOC11
SC17	SRR5803168	Anhui white goat	SC_LOC12
SC18	SRR5803178	Shannan White goat	SC_LOC13

Table S6. Summary of candidate selective sweeps.

Pop	Chr	Start	End	F_{ST}	ln($\theta\pi$_SC / $\theta\pi$_NC)	Genes	Nearest Gene
NC	3	95320001	95380000	0.244	0.818	SPAG17	
NC	5	22830001	22980000	0.395	1.230	NUDT4	
NC	6	95400001	95630000	0.530	1.149	C6H4orf22, FGF5, TRNAG-CCC	
NC	6	95670001	95790000	0.297	0.785	C6H4orf22	
NC	15	39470001	39520000	0.252	0.275	SBF2	
NC	18	16060001	16120000	0.243	1.266	LOC102181148, SPIRE2, TCF25	
NC	18	51810001	51890000	0.250	0.427	CIC, MEGF8, PAFAH1B3, PRR19, TMEM145	
NC	19	47010001	47060000	0.258	0.491	TANC2	
NC	X1	14150001	14650000	0.528	1.362	MAGED1	
NC	X1	17890001	18490000	0.621	1.400	EDA2R	
SC	3	44140001	44200000	0.331	-0.975	-	DEPDC1
SC	3	96520001	96720000	0.412	-1.563	WARS2	
SC	4	86940001	87000000	0.368	-0.954	KIAA1324L	
SC	5	18440001	18500000	0.248	-1.103	-	TRNAC-GCA
SC	5	64460001	64570000	0.283	-0.894	CCDC53, NUP37	
SC	6	70620001	70690000	0.256	-0.618	-	KIT
SC	7	670001	750000	0.293	-1.986	LOC102186959	
SC	18	25880001	25930000	0.249	-0.368	-	GNAO1
SC	19	33640001	33690000	0.246	-0.595	SLC47A1	
SC	21	23130001	23180000	0.249	-0.633	-	MEX3B
SC	22	51690001	51780000	0.301	-1.577	CDC25A,LOC102170003,LOC102171106,MAP28	
SC	26	30490001	30540000	0.240	-0.427	BLOC1S2, CHUK, CWF19L1	
SC	26	30550001	30620000	0.257	-0.503	CHUK,ERLIN1,LOC102175359,LOC102180722	
SC	X1	18470001	18530000	0.457	-0.659	EDA2R	

NOTE: NW_017189516.1 is referred to as X1. In each NC-selected region, maximum F_{ST} and maximum ln(θπ_SC / θπ_NC) within 50-kbp windows is shown. In each SC-selected region, maximum F_{ST} and minimum ln(θπ_SC / θπ_NC) within 50-kbp windows is shown.

Table S7. Summary of *D*-statistics.

H1	H2	H3	H4	JK-D	Z	pvalue
Neolithic_East	Neolithic_Levant	Shimao	argali	-0.2260	-10.575	<0.000001
Neolithic_East	Neolithic_West	Shimao	argali	-0.1018	-17.135	<0.000001
Neolithic_East	Neolithic_Levant	Muzhuzhuliang	argali	-0.2281	-35.768	<0.000001
Neolithic_East	Neolithic_West	Muzhuzhuliang	argali	-0.0779	-29.884	<0.000001
Neolithic_East	Neolithic_Levant	Xiaoshuanggucheng	argali	-0.2199	-29.708	<0.000001
Neolithic_East	Neolithic_West	Xiaoshuanggucheng	argali	-0.0795	-25.403	<0.000001
Neolithic_East	Neolithic_Levant	Xinzhouyaozi	argali	-0.2144	-29.088	<0.000001
Neolithic_East	Neolithic_West	Xinzhouyaozi	argali	-0.0853	-30.371	<0.000001
Neolithic_East	Neolithic_Levant	Wangdahu	argali	-0.2340	-60.644	<0.000001
Neolithic_East	Neolithic_West	Wangdahu	argali	-0.0859	-35.309	<0.000001
Neolithic_East	Neolithic_Levant	Jiulongshan	argali	-0.2466	-17.628	<0.000001
Neolithic_East	Neolithic_West	Jiulongshan	argali	-0.0810	-19.503	<0.000001
Neolithic_East	Neolithic_Levant	Zhongzhuang	argali	-0.1771	-6.258	<0.000001
Neolithic_East	Neolithic_West	Zhongzhuang	argali	-0.0863	-10.716	<0.000001
Neolithic_East	Neolithic_Levant	Yanjialiang	argali	-0.2275	-60.157	<0.000001
Neolithic_East	Neolithic_West	Yanjialiang	argali	-0.0861	-38.777	<0.000001
Neolithic_East	Neolithic_Levant	Tianxi	argali	-0.2399	-53.600	<0.000001
Neolithic_East	Neolithic_West	Tianxi	argali	-0.0959	-41.929	<0.000001
Neolithic_East	Neolithic_Levant	NC	argali	-0.2395	-73.556	<0.000001
Neolithic_East	Neolithic_West	NC	argali	-0.0952	-53.385	<0.000001
Neolithic_East	Neolithic_Levant	SC	argali	-0.2575	-76.489	<0.000001
Neolithic_East	Neolithic_West	SC	argali	-0.1110	-56.962	<0.000001
Chalcolithic_Iran	Neolithic_East	Shimao	argali	-0.0311	-3.391	0.000697
Chalcolithic_Iran	BronzeAge_Iran	Shimao	argali	-0.0226	-2.071	0.038343
Chalcolithic_Iran	Neolithic_East	Muzhuzhuliang	argali	-0.0293	-8.499	<0.000001
Chalcolithic_Iran	BronzeAge_Iran	Muzhuzhuliang	argali	-0.0080	-2.059	0.039510
Chalcolithic_Iran	Neolithic_East	Xiaoshuanggucheng	argali	-0.0263	-6.976	<0.000001
Chalcolithic_Iran	BronzeAge_Iran	Xiaoshuanggucheng	argali	-0.0109	-2.425	0.015327
Chalcolithic_Iran	Neolithic_East	Xinzhouyaozi	argali	-0.0268	-7.221	<0.000001
Chalcolithic_Iran	BronzeAge_Iran	Xinzhouyaozi	argali	-0.0134	-2.970	0.002982
Chalcolithic_Iran	Neolithic_East	Wangdahu	argali	-0.0265	-9.775	<0.000001
Chalcolithic_Iran	BronzeAge_Iran	Wangdahu	argali	-0.0110	-3.534	0.000410
Chalcolithic_Iran	Neolithic_East	Jiulongshan	argali	-0.0249	-4.118	0.000038
Chalcolithic_Iran	BronzeAge_Iran	Jiulongshan	argali	-0.0173	-2.412	0.015883
Chalcolithic_Iran	Neolithic_East	Zhongzhuang	argali	-0.0228	-1.865	0.062230
Chalcolithic_Iran	BronzeAge_Iran	Zhongzhuang	argali	-0.0120	-0.801	0.423113
Chalcolithic_Iran	Neolithic_East	Yanjialiang	argali	-0.0232	-9.465	<0.000001
Chalcolithic_Iran	BronzeAge_Iran	Yanjialiang	argali	-0.0049	-1.708	0.087727
Chalcolithic_Iran	Neolithic_East	Tianxi	argali	-0.0232	-8.737	<0.000001
Chalcolithic_Iran	BronzeAge_Iran	Tianxi	argali	-0.0088	-2.881	0.003965
Chalcolithic_Iran	Neolithic_East	NC	argali	-0.0192	-9.602	<0.000001
Chalcolithic_Iran	BronzeAge_Iran	NC	argali	-0.0061	-2.694	0.007056
Chalcolithic_Iran	Neolithic_East	SC	argali	-0.0159	-6.618	<0.000001
Chalcolithic_Iran	BronzeAge_Iran	SC	argali	-0.0126	-4.886	0.000001
NC	SC	EUR	argali	-0.0203	-17.297	<0.000001
NC	SC	AFR	argali	-0.0172	-14.285	<0.000001
NC	SC	SWA	argali	-0.0059	-5.410	<0.000001

NC	SC	SAS	argali	0.0107	8.150	<0.000001
NC	Shimao	EUR	argali	-0.0147	-2.503	0.012321
NC	Shimao	AFR	argali	-0.0078	-1.335	0.181901
NC	Shimao	SWA	argali	-0.0021	-0.400	0.689199
NC	Shimao	SAS	argali	0.0077	1.408	0.159215
SC	Shimao	EUR	argali	0.0020	0.325	0.745078
SC	Shimao	AFR	argali	0.0049	0.797	0.425460
SC	Shimao	SWA	argali	-0.0001	-0.012	0.990427
SC	Shimao	SAS	argali	-0.0074	-1.299	0.193942

Table S8. Posterior probabilities of parameters in demographic Model 3 (Fig. S16). Time is in unit of generations.

Parameter	Average	Median	Mode	95% HPD
N1	4,700	1,620	329	104~32,300
N2	3,290	2,100	1,360	648~15,800
N3	42,400	44,000	47,600	14,400~58,800
N4	35,300	35,700	30,300	8,520~58,900
t0	269	242	210	33~647
ra	3.64%	2.64%	1.97%	0.68%~13.10%
N1b	47,500	48,300	49,700	29,600~58,900
t1	1,220	1,220	1,250	780~1,640
N2b	35,300	35,900	37,500	13,500~54,600
tA	6,380	6,260	4,940	3,360~9,750
NA	37,700	38,300	36,700	17,500~56,500

References

Bickhart DM, Rosen BD, Koren S, Sayre BL, Hastie AR, Chan S, Lee J, Lam ET, Liachko I, Sullivan ST, et al. 2017. Single-molecule sequencing and chromatin conformation capture enable de novo reference assembly of the domestic goat genome. *Nat. Genet.* 49:643–650.

Briggs AW, Good JM, Green RE, Krause J, Maricic T, Stenzel U, Lalueza-Fox C, Rudan P, Brajkovic D, Kucan Z, et al. 2009. Targeted retrieval and analysis of five Neandertal mtDNA genomes. *Science* 325:318–321.

Browning B, Browning S. 2016. Genotype Imputation with Millions of Reference Samples. *Am J Hum Genet* 98:116.

Browning SR, Browning BL. 2007. Rapid and accurate haplotype phasing and missing-data inference for whole-genome association studies by use of localized haplotype clustering. *Am. J. Hum. Genet.* 81:1084–1097.

Casper J, Zweig AS, Villarreal C, Tyner C, Speir ML, Rosenbloom KR, Raney BJ, Lee CM, Lee BT, Karolchik D, et al. 2017. the UCSC Genome Browser database: 2018 update. *Nucleic Acids Res.* 46:D762–D769.

Chen N, Cai Y, Chen Q, Li R, Wang K, Huang Y, Hu S, Huang S, Zhang H, Zheng Z, et al. 2018. Whole-genome resequencing reveals world-wide ancestry and adaptive introgression events of domesticated cattle in East Asia. *Nat. Commun.* 9:2337.

Chen Q, Cao J. 2006. A Research of Funerary Animals of Bancheng Graveyard in Liangcheng County Inner Mongolia. *Res. China's Front. Archaeol.* 0:25. (in Chinese)

Chen S, Zhou Y, Chen Y, Gu J. 2018. fastp: an ultra-fast all-in-one FASTQ preprocessor. *Bioinformatics* 34:i884–i890.

Cornuet JM, Pudlo P, Veyssier J, Dehne-Garcia A, Gautier M, Leblois R, Marin JM, Estoup A. 2014. DIYABC v2.0: A software to make approximate Bayesian computation inferences about population history using single nucleotide polymorphism, DNA sequence and microsatellite data. *Bioinformatics* 30:1187–1189.

Daly KG, Maisano Delser P, Mullin VE, Scheu A, Mattiangeli V, Teasdale MD, Hare AJ, Burger J, Verdugo MP, Collins MJ, et al. 2018. Ancient goat genomes reveal mosaic domestication in the Fertile Crescent. *Science* 361:85–88.

Danecek P, Auton A, Abecasis G, Albers CA, Banks E, Depristo MA, Handsaker RE, Lunter G, Marth GT, Sherry ST. 2011. The variant call format and VCFtools. *Bioinformatics* 27:2156–2158.

Edgar RC. 2004. MUSCLE: multiple sequence alignment with high accuracy and high throughput. *Nucleic Acids Res.* 32:1792–1797.

Gutenkunst RN, Hernandez RD, Williamson SH, Bustamante CD. 2009. Inferring the Joint Demographic History of Multiple Populations from Multidimensional SNP Frequency Data.McVean G, editor. *PLoS Genet.* 5:e1000695.

Handsaker RE, Van Doren V, Berman JR, Genovese G, Kashin S, Boettger LM, McCarroll SA. 2015. Large multiallelic copy number variations in humans. *Nat. Genet.* 47:296–303.

Hu S, Yang M, Song Z, Shao J. 2016. 2012-2013 nian du Shaanxi Shenmu Shimao yizhi chutu dongwu yicun yanjiu. *Kaogu yu Wenwu* 4:109–121. (in Chinese)

Jian Z, Xiao-ni L, Zhong-hou T, Quan-gong C. 2012. Mapping of the north-south demarcation zone in China based on GIS. *J. Lanzhou Univ. (Natural Sciences).* 48:28–33. (in Chinese)

Jónsson H, Ginolhac A, Schubert M, Johnson PLF, Orlando L. 2013. mapDamage2. 0: fast approximate Bayesian estimates of ancient DNA damage parameters. *Bioinformatics* 29:1682–1684.

Kent WJ. 2002. BLAT--the BLAST-like alignment tool. *Genome Res.* 12:656–664.

Kent WJ, Sugnet CW, Furey TS, Roskin KM, Pringle TH, Zahler AM, Haussler D. 2002. The human genome browser at UCSC. *Genome Res.* 12:996–1006.

Kochen C. 1973. A Preliminary Study on the Climatic Fluctuation during the last 5000 years in China. *Sci. Sin.*:226–256.

Korneliussen TS, Albrechtsen A, Nielsen R. 2014. ANGSD: analysis of next generation sequencing data. *BMC Bioinformatics* 15:356.

Kruschke JK. 2013. Bayesian estimation supersedes the t test. *J. Exp. Psychol. Gen.* 142:573–603.

Kumar S, Stecher G, Li M, Knyaz C, Tamura K. 2018. MEGA X: Molecular Evolutionary Genetics Analysis across Computing Platforms. *Mol. Biol. Evol.* 35:1547–1549.

Kumar S, Stecher G, Suleski M, Hedges SB. 2017. TimeTree: A Resource for Timelines, Timetrees, and Divergence Times. *Mol. Biol. Evol.* 34:1812–1819.

Lawson DJ, Hellenthal G, Myers S, Falush D. 2012. Inference of population structure using dense haplotype data. *PLoS Genet* 8:e1002453.

Letunic I, Bork P. 2016. Interactive tree of life (iTOL) v3: an online tool for the display and annotation of phylogenetic and other trees. *Nucleic Acids Res.* 44:W242–W245.

Li H. 2013. Aligning sequence reads, clone sequences and assembly contigs with BWA-MEM. *arXiv Prepr. arXiv1303.3997*.

Li H, Durbin R. 2010. Fast and accurate long-read alignment with Burrows–Wheeler transform. *Bioinformatics* 26:589–595.

Li H, Handsaker B, Wysoker A, Fennell T, Ruan J, Homer N, Marth G, Abecasis G, Durbin R, 1000 Genome Project Data Processing Subgroup. 2009. The Sequence Alignment/Map format and SAMtools. *Bioinformatics* 25:2078–2079.

Liu Z, Yang F, Zhao M, Mu Q, Che T, Li J, Zhao Y. 2019. Skin transcriptome reveals the Periodic changes in genes underlying hair follicle cycling in Cashmere. *bioRxiv*:554030.

Luo F. 2018. The Animal Sacrifice Burial Custom of the North System Bronze Cultures of China. *Acta Archaeol. Sin.*:183–200. (in Chinese)

Owlett TE, Hu S, Sun Z, Shao J. 2018. Food between the country and the city: The politics of food production at Shimao and Zhaimaoliang in the Ordos Region, northern China. *Archaeol. Res. Asia* 14:46–60.

Paradis E. 2010. pegas: an R package for population genetics with an integrated-modular approach. *Bioinformatics* 26:419–420.

Patterson N, Moorjani P, Luo Y, Mallick S, Rohland N, Zhan Y, Genschoreck T, Webster T, Reich D. 2012. Ancient admixture in human history. *Genetics* 192:1065–1093.

Patterson N, Price AL, Reich D. 2006. Population Structure and Eigenanalysis. *PLoS Genet.* 2:e190.

Pedregosa F, Varoquaux G, Gramfort A, Michel V, Thirion B, Grisel O, Blondel M, Prettenhofer P, Weiss R, Dubourg V, et al. 2011. Scikit-learn: Machine Learning in Python. *J. Mach. Learn. Res.* 12:2825–2830.
Pickrell JK, Pritchard JK. 2012. Inference of population splits and mixtures from genome-wide allele frequency data. *PLoS Genet* 8:e1002967.
Poplin R, Ruano-Rubio V, DePristo MA, Fennell TJ, Carneiro MO, Auwera GA Van der, Kling DE, Gauthier LD, Levy-Moonshine A, Roazen D, et al. 2018. Scaling accurate genetic variant discovery to tens of thousands of samples. *bioRxiv*:201178.
Purcell S, Neale B, Todd-Brown K, Thomas L, MA F, Bender D, Maller J, Sklar P, PI de B, MJ D, et al. 2007. PLINK: A Tool Set for Whole-Genome Association and Population-Based Linkage Analyses. *Am J Hum Genet* 81:559–575.
Rosenbloom KR, Sloan CA, Malladi VS, Dreszer TR, Learned K, Kirkup VM, Wong MC, Maddren M, Fang R, Heitner SG, et al. 2012. ENCODE Data in the UCSC Genome Browser: year 5 update. *Nucleic Acids Res.* 41:D56–D63.
Salvatier J, Wiecki T V., Fonnesbeck C. 2016. Probabilistic programming in Python using PyMC3. *PeerJ Comput. Sci.* 2016.
Soraggi S, Wiuf C, Albrechtsen A. 2018. Powerful Inference with the D-Statistic on Low-Coverage Whole-Genome Data. *G3 (Bethesda).* 8:551–566.
Sun Z, Shao J, Cui J, Bonomo MF, Guo Q, Wu X, Wang J. 2018. The first Neolithic urban center on China's north Loess Plateau: The rise and fall of Shimao. *Archaeol. Res. Asia* 14:33–45.
Sun Z, Shao J, Shao A, Kang N, Qu F, Bai H. 2015. Shaanxi Shenmu xian Shimao yizhi Houyangwan, Hujiawa di dian shi jue jian bao (The Trial Excavations of the Houyangwan and Hujiawa Localities of the Shimao Site in Shenmu County, Shanxi). *Kaogu* 5:62–71. (in Chinese)
Ta L, Zhang Haibing, Zhang Hongxing. 2010. Excavation Report of the Yanjialiang Archaeological Site in Baotou. Beijing: Science Press. (in Chinese)
Wang W, Guo X, Kang N, Liu X, Hu K, Chen L. 2015. Preliminary Report on the Excavation of Muzhuzhuliang Site in Shenmu,Shaanxi. *Archaeol. Cult. Reli.*:3–11. (in Chinese)
Yang DY, Eng B, Waye JS, Dudar JC, Saunders SR. 1998. Improved DNA extraction from ancient bones using silica-based spin columns. *Am. J. Phys. Anthropol.* 105:539–543.
Zhao Y Bin, Li HJ, Cai DW, Li CX, Zhang QC, Zhu H, Zhou H. 2010. Ancient DNA from nomads in 2500-year-old archeological sites of Pengyang, China. *J. Hum. Genet.* 55:215–218.
Zheng Z, Wang Xihong, Li M, Li Y, Yang Z, Wang Xiaolong, Pan X, Gong M, Zhang Y, Guo Y, et al. 2020. The origin of domestication genes in goats. *bioRxiv*:2020.01.14.905505.
